# Metformin mitigates SASP secretion and LPS-triggered hyper-inflammation in Doxorubicin-induced senescent endothelial cells

**DOI:** 10.3389/fragi.2023.1170434

**Published:** 2023-04-24

**Authors:** Ibrahim Y. Abdelgawad, Kevin Agostinucci, Bushra Sadaf, Marianne K. O. Grant, Beshay N. Zordoky

**Affiliations:** Department of Experimental and Clinical Pharmacology, University of Minnesota College of Pharmacy, Minneapolis, MN, United States

**Keywords:** endothelial senescence, doxorubicin, metformin, SASP, LPS, senomorphics

## Abstract

**Introduction:** Doxorubicin (DOX), a chemotherapeutic drug, induces senescence and increases the secretion of senescence-associated secretory phenotype (SASP) in endothelial cells (ECs), which contributes to DOX-induced inflammaging. Metformin, an anti-diabetic drug, demonstrates senomorphic effects on different models of senescence. However, the effects of metformin on DOX-induced endothelial senescence have not been reported before. Senescent ECs exhibit a hyper-inflammatory response to lipopolysachharide (LPS). Therefore, in our current work, we identified the effects of metformin on DOX-induced endothelial senescence and LPS-induced hyper-inflammation in senescent ECs.

**Methods:** ECs were treated with DOX ± metformin for 24 h followed by 72 h incubation without DOX to establish senescence. Effects of metformin on senescence markers expression, SA-β-gal activity, and SASP secretion were assessed. To delineate the molecular mechanisms, the effects of metformin on major signaling pathways were determined. The effect of LPS ± metformin was determined by stimulating both senescent and non-senescent ECs with LPS for an additional 24 h.

**Results:** Metformin corrected DOX-induced upregulation of senescence markers and decreased the secretion of SASP factors and adhesion molecules. These effects were associated with a significant inhibition of the JNK and NF-κB pathway. A significant hyper-inflammatory response to LPS was observed in DOX-induced senescent ECs compared to non-senescent ECs. Metformin blunted LPS-induced upregulation of pro-inflammatory SASP factors.

**Conclusion:** Our study demonstrates that metformin mitigates DOX-induced endothelial senescence phenotype and ameliorates the hyper-inflammatory response to LPS. These findings suggest that metformin may protect against DOX-induced vascular aging and endothelial dysfunction and ameliorate infection-induced hyper-inflammation in DOX-treated cancer survivors.

## 1 Introduction

Doxorubicin (DOX) is a chemotherapeutic drug used for the treatment of solid and blood cancers (Chatterjee et al., 2010). Although DOX has contributed to improved survival rates in cancer patients, these survivors experience premature aging and frailty (Armenian et al., 2019). DOX induces premature aging primarily by accumulated DNA damage due to inhibition of topoisomerase II (Zhang et al., 2012) and increased reactive oxygen species (ROS) resulting from mitochondrial dysfunction (Asensio-Lopez et al., 2017). Both mechanisms can initiate a signaling cascade that prevents cells from undergoing replication, termed senescence.

DOX induces senescence in different cardiovascular cells, including cardiomyocytes, endothelial cells (ECs), cardiac fibroblasts, and cardiac progenitor cells (Abdelgawad et al., 2020). However, a landmark study demonstrated that, following DOX administration in p16-3MR male mice, the majority of senescent cardiac cells were ECs (Demaria et al., 2017). Moreover, a recent study by Yousefzadeh et al. (2020) used an accelerated aging mouse model and screened the expression of the senescence markers p21 and p16 in different tissues. Interestingly, the aorta demonstrated the highest expression of p16 and the second highest expression of p21 compared to other organs, with no significant increase in the expression of these markers in the heart (Yousefzadeh et al., 2020). Collectively, these studies suggest that ECs are a salient target for the induction of senescence, and that endothelial senescence plays a major role in DOX-induced cardiovascular complications.

Endothelial senescence is associated with multiple cellular and functional alterations that contribute to endothelial dysfunction, including impairment of vascular permeability, altered angiogenic response, and decreased endothelium-dependent dilation (Lesniewski et al., 2017). Importantly, senescent ECs secrete pro-inflammatory cytokines and metalloproteases known as senescence-associated secretory phenotype (SASP) (Abdelgawad et al., 2020). Accumulation of SASP promotes chronic low-grade inflammation, known as “inflammaging” (Franceschi et al., 2000), which can affect endothelial function (Soysal et al., 2020). Indeed, vascular senescence has been identified as a significant contributor to multiple cardiovascular diseases [reviewed in (Katsuumi et al., 2018; Jia et al., 2019)]. Furthermore, cancer survivors treated with anthracyclines exhibit endothelial dysfunction and vascular damage (Terwoord et al., 2022), which can be partially attributed to endothelial senescence as we recently reviewed (Abdelgawad et al., 2022b). Therefore, there is a compelling need for pharmacological strategies that target endothelial senescence to preserve endothelial function and potentially mitigate the related adverse effects in cancer survivors.

The hypothesis that senescent cells contribute to the pathogenesis of age-related diseases has led to the development of a new class of drugs called senotherapeutics (Baker et al., 2011). Senotherapeutics can be divided into two categories: senolytics and senomorphics, both of which mitigate senescence. Senolytics induce apoptosis and selectively eliminate senescent cells (Tse et al., 2008), whereas senomorphics modulate the secretion of SASP from senescent cells, thereby improving cellular functions (Moiseeva et al., 2013; Laberge et al., 2015).

Metformin, a widely used drug for the treatment of type 2 diabetes, was recently demonstrated to exert senomorphic and anti-aging effects (Chen et al., 2022b; Khodadadi et al., 2022). These effects are mediated by the ability of metformin to reduce ROS levels (Acar et al., 2021) and prevent DNA double-strand breaks (Park et al., 2022). Metformin has also been shown to have anti-inflammatory effects as evidenced by its ability to suppress SASP secretion in IMR90 fibroblasts (Moiseeva et al., 2013), bronchial-alveolar epithelial cells (Hansel et al., 2021; Wang et al., 2021), and lens epithelial cells (Chen et al., 2022a). Additionally, metformin has been shown to protect against endothelial senescence in different models of senescence including radiation- (Park et al., 2022), lipopolysaccharide (LPS)- (Raj et al., 2021), and high glucose-induced senescence (Arunachalam et al., 2014; Zhang et al., 2015). However, the effects of metformin on DOX-induced endothelial senescence have not been reported. Therefore, the current study aims to identify the senomorphic effects of metformin against DOX-induced endothelial senescence.

In recent years, there has been growing interest in the role of senescent ECs in LPS-induced inflammation, as ECs are of the major cellular targets of LPS-induced inflammation. Newly arising evidence show that radiation-induced and replicative senescent ECs are more vulnerable to LPS-induced inflammation than non-senescent ECs (Suzuki et al., 2019; Camell et al., 2021). Given that a significant percentage of cancer survivors received DOX during their treatment, we determined the effect of LPS stimulation on DOX-induced senescent ECs and identified the effects of metformin on LPS-induced hyper-inflammation.

## 2 Materials and methods

### 2.1 Cell culture

Human umbilical vein endothelial cells (HUVECs) and EA.hy926 human endothelial-derived cell lines were purchased from American Type Culture Collection (ATCC, Manassas, FL, United States). HUVECs were cultured in vascular cell basal medium (ATCC) supplemented with endothelial cell growth kit–VEGF, including 5 ng/mL rh VEGF, 5 ng/mL rh EGF, 5 ng/mL rh FGF basic, 15 ng/mL rh IGF, 10 mM L-glutamine, 0.75 U/mL heparin sulfate, hydrocortisone 1 μg/mL, ascorbic acid 50 μg/mL, fetal bovine serum 2%, 10 U/mL penicillin, and 10 μg/mL streptomycin. EA.hy926 cells were cultured in Dulbecco’s modified Eagle’s medium (DMEM) supplemented with 10% (v/v) fetal bovine serum, 100 U/mL penicillin, and 100 μg/mL streptomycin (MilliporeSigma, St. Louis, MO, United States). Both cell lines were incubated at 37°C in 75 cm^2^ tissue culture-treated flasks in a 5% CO_2_ humidified incubator. Every other day, the media were replaced and the cells were subcultured at 80% confluence.

### 2.2 Cell treatments

Both EA.hy926 cells and HUVECs were pretreated for 24 h with increasing concentrations of metformin (0.5 mM, 1 mM, 2 mM, and 5 mM for EA.hy926 cells or 2 mM and 5 mM for HUVECs). Then, cells were co-treated with DOX and metformin for an additional 24 h. Based on our previous study (Abdelgawad et al., 2022a), the clinically-relevant concentration 0.5 μM of DOX was selected to induce senescence in ECs since this concentration was associated with highest induction of senescence markers. Thereafter, cells were washed with PBS to remove DOX, metformin was added back to the medium, and cells were incubated for a further 72 h for protein extraction or 120 h for SA-β-gal staining.

For LPS experiments, HUVECs were either treated with DOX for 24 h, followed by 72 h incubation without DOX to establish senescence, or left untreated as non-senescent cells. The effect of LPS was then determined by stimulating both senescent and non-senescent HUVECs with LPS (30 ng/mL) for an additional 24 h. Notably, the media were changed before adding LPS so that the assessed SASP factors in the media reflect mainly the response of control and senescent ECs to LPS. Metformin was added as described above to determine its effect on LPS stimulation.

DOX, metformin, and LPS were purchased from Sigma (St. Louis, MO, United States) and stock solutions were prepared by dissolving them in the corresponding media of each cell line. All the cell treatments were performed between passages 5 and 10 in EA.hy926 cells and between passages 3 and 6 in HUVECs.

### 2.3 Protein extraction and western blotting

Following the treatments described above, EA.hy926 cells and HUVECs were washed twice with PBS and harvested in lysis buffer containing 20 mM Tris, 10 mM sodium pyrophosphate, 100 mM sodium fluoride, 5 mM EDTA, and 1% NP-40 supplemented with protease and phosphatase inhibitors. Cells were passed through a 28 gauge needle 10 times to further lyse the cells. Thereafter, the cell lysate was centrifuged at 2,000 x g for 10 min at 4°C and the supernatant was collected for western blotting. Protein concentration was measured using Pierce™ bicinchoninic acid (BCA) protein assay kit according to manufacturer’s instructions (Thermo Fisher Scientific, Waltham, MA, United States). Cell homogenates were denatured by boiling at 100°C for 5 min in sodium dodecyl sulfate (SDS)-polyacrylamide gel electrophoresis (PAGE) loading buffer (G Biosciences, St. Louis, MO, United States) containing 20 mM dithiothreitol. Thereafter, 20 μg homogenates were separated on 8%, 12%, or 15% SDS-PAGE gels and electrophoretically transferred to nitrocellulose membranes. The blots were then blocked at room temperature for 1 h using a blocking buffer consisting of 5% skim milk powder in Tris-buffered saline (20 mM Tris, 150 mM NaCl, pH 7.4) with 0.05% (v/v) Tween-20 (TBST). Following blocking, blots were incubated overnight at 4°C with primary antibodies diluted in 1% milk solution in TBST. Blots were then washed in TBST and incubated for 1 h at room temperature with horseradish peroxidase (HRP)-conjugated secondary antibodies diluted in blocking buffer, then washed with TBST. Blots were visualized using Pierce™ ECL substrate (Thermo Fisher Scientific) according to the manufacturer’s instructions. Primary mouse antibodies against p53 (catalog 2,524, 1:1000 dilution) and primary rabbit antibodies against phospho-p53 (Ser15) (catalog 9284, 1:1000 dilution), p21 (catalog 2,947, 1:1000 dilution), MMP-3 (catalog 14,351, 1:1000 dilution), ICAM-1 (catalog 4915, 1:1000 dilution), phospho-SAPK/JNK (Thr183/Tyr185) (catalog 4668, 1:1000 dilution), SAPK/JNK (catalog 9252, 1:1000 dilution), phospho-p38 (Thr180/Tyr182) (catalog 4511, 1:1000 dilution), p38 (catalog 8690, 1:1000 dilution), AMPK alpha (catalog 2,532, 1:1000 dilution), phospho-NF-κB p65 (catalog 3033, 1:1000 dilution), and alpha-tubulin (catalog 2144, 1:1000 dilution) were purchased from Cell Signaling Technology (Danvers, MA, United States). Primary rabbit antibodies against phospho-AMPK alpha (Thr172) (catalog 07–681, 1:1000 dilution) were purchased from MilliporeSigma (Burlington, MA, United States). HRP-conjugated horse anti-mouse secondary antibodies were purchased from Cell Signaling (catalog 7076; 1:1000 dilution) and HRP-conjugated goat anti-rabbit secondary antibodies were purchased from Jackson ImmunoResearch (catalog 111–035–144, West Grove, PA, United States; 1:10,000 dilution). ImageJ software (National Institutes of Health, Bethesda, MD, United States) was used to quantify band intensities using alpha-tubulin protein levels as normalizing loading controls. Phospho-protein band intensities were measured relative to the respective total protein level. In some experiments, the blots were cut at separate molecular weight marks, thereby allowing the same blot to be incubated with more than one primary antibody at the same time.

### 2.4 Senescence associated-B-galactosidase (SA-β-gal) assay

For the detection of senescence, the SA-β-gal staining kit (Cell Signaling Technology) was used to stain senescent cells according to the manufacturer’s protocol. Incubation time and pH were optimized as previously described (Abdelgawad et al., 2022a). To calculate the percentage of cells that were positive for SA-β-gal, the number of stained cells was counted relative to the total number of cells (at least 100 cells) using a bright-field microscope with a ×4 objective lens.

### 2.5 Assessment of senescence-associated secretory phenotype (SASP) factors in cell culture media

After the specified treatments, the media from HUVECs was collected and stored at −80°C until use. The supernatants were analyzed by the Cytokine Reference Laboratory at the University of Minnesota for the detection of human-specific interleukin 6 (IL-6), tumor necrosis factor-alpha (TNF-α), macrophage inflammatory protein-1 alpha (MIP-1α), monocyte chemoattractant protein-3 (MCP-3), monocyte chemoattractant protein-1 (MCP-1), C-X-C motif chemokine ligand 1 (CXCL1), C-X-C motif chemokine ligand 2 (CXCL2), interleukin-1 beta (IL-1β), interleukin 8 (IL-8), matrix metalloproteinase-3 (MMP-3), intracellular adhesion molecule-1 (ICAM-1), and E-selectin using the Luminex multiplex platform. The cytokines were analyzed according to the manufacturer’s guidelines by lab personnel who were unaware of the experimental design. Samples were run in duplicate and the values were interpolated from 5-parameter-equipped standard curves. Cytokine concentrations were reported after being normalized to the protein content of the cells, which was determined by BCA.

### 2.6 Statistical analysis

Data analysis was performed using GraphPad Prism software (version 8.3.0, La Jolla, CA, www.graphpad.com) and the data are presented as mean ± standard error of the mean (SEM). Normality was checked using the Shapiro-Wilk test. Comparisons between control, DOX, and metformin treatments were performed using a one-way analysis of variance (ANOVA) followed by pair-wise comparisons relative to DOX treatment using Dunnet’s multiple comparison test (if the normality test was passed) or non-parametric Kruskal–Wallis tests followed by Dunn’s *post hoc* test (if the normality test was failed). For LPS experiments (Figures 5, 6), comparisons were performed by ordinary two-way analysis of variance (ANOVA), followed by Tukey’s multiple comparison *post hoc* analysis. A *p*-value of <0.05 was chosen to indicate statistical significance.

## 3 Results

### 3.1 Metformin inhibits DOX-upregulated expression of senescence markers in ECs

**FIGURE 1:**
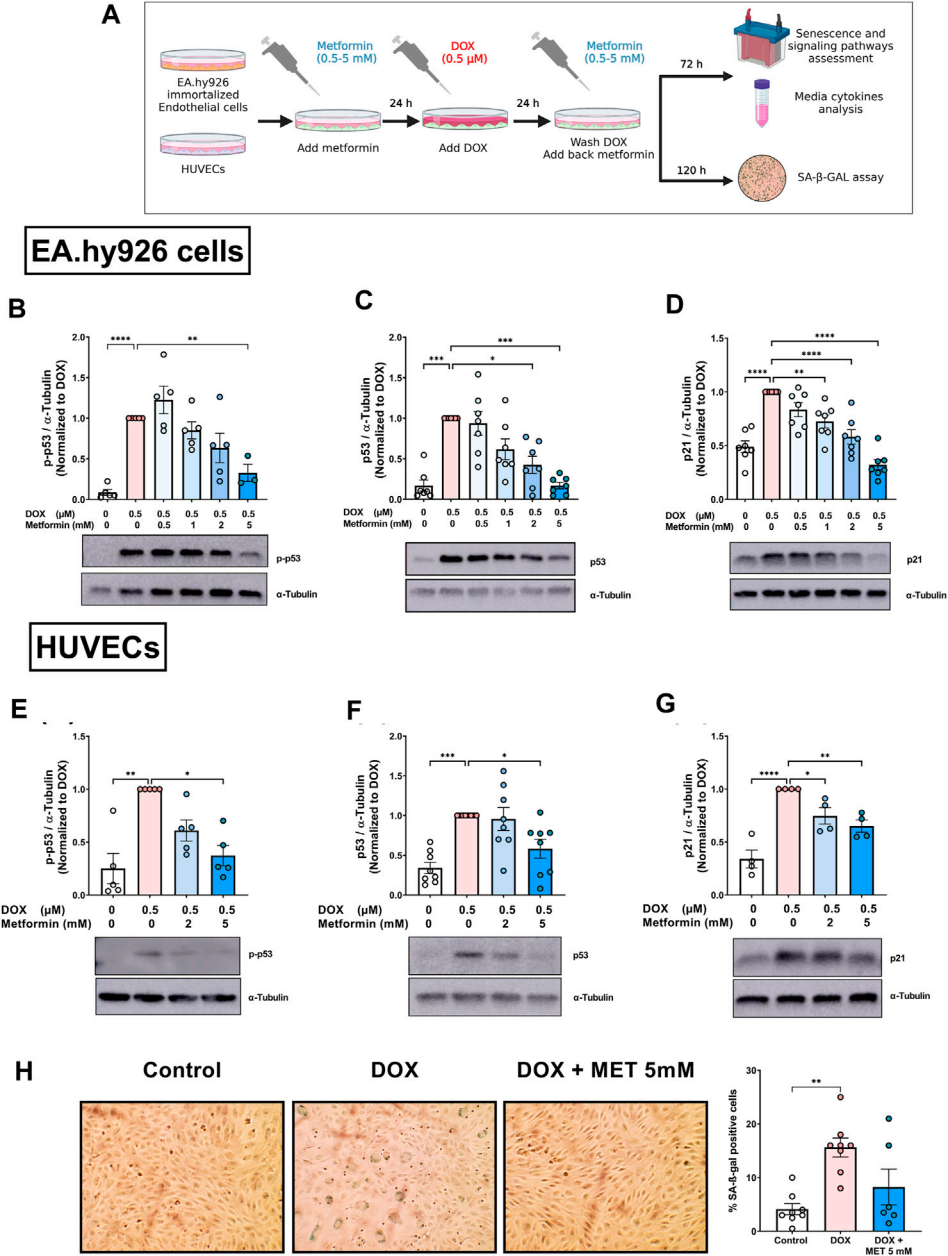
Metformin inhibited DOX-induced upregulation of senescence markers and SA-β-gal activity in endothelial cells. **(A)** Schematic diagram of the experimental design. Both EA.hy926 endothelial derived cells and HUVECs were treated for 24 h with 0.5 µM DOX ± metformin (0.5–5 mM, added 24 h before DOX). Thereafter, DOX was removed and the cells were incubated in DOX-free media with or without metformin for an additional 72 h for protein expression experiments or 120 h for measurement of SA-β-gal staining. Expression levels of senescence markers including p-p53, p53, and p21 in EA.hy926 cells **(B–D),** and HUVECs [**(E–G)**, respectively] were measured using western blot (n = 4–8). Representative images of western blots are shown. Values were normalized to α-tubulin and expressed relative to cells treated with DOX alone. **(H)** Images of SA-β-gal staining in control, DOX-treated, and DOX + metformin co-treated cells are shown in HUVECs. Images were analyzed and the percentage of SA-β-gal positive cells were calculated (n = 6–8). Values are presented as means ± SEM. Data were analyzed by one-way ANOVA followed by a Dunnet’s multiple comparisons test (Figures 1B,D,F–H) or non-parametric Kruskal–Wallis tests followed by Dunn’s *post hoc* test (Figures 1C, E); * *p* < 0.05, ** *p* < 0.01, *** *p* < 0.001, **** *p* < 0.0001. Schematic diagram created with BioRender.com.

Previously, we characterized DOX-induced senescence phenotype in two types of endothelial cells: immortalized EA.hy926 endothelial-derived cells and primary human umbilical vein endothelial cells (HUVECs) (Abdelgawad et al., 2022a). However, the effect of metformin on DOX-induced endothelial senescence phenotype has not been reported yet. In this study, we first evaluated the effects of metformin in EA.hy926 endothelial-derived cells. Cells were treated with 0.5 µM DOX with or without metformin in a concentration range of 0.5–5 mM as illustrated in Figure 1A. The senescence phenotype was evaluated by measuring the protein expression of senescence markers p-p53, p53, and p21. DOX alone upregulated all the assessed senescence markers including p-p53 (Figure 1B), p53 (Figure 1C), and p21 (Figure 1D) by 11.5-, 5.9-, and 2-fold, respectively. These markers were expected to be upregulated by DOX since the p53/p21 pathway is activated in response to DNA damage (Abdelgawad et al., 2022a). Importantly, pretreatment with the highest concentration of metformin (5 mM) downregulated the phosphorylation of p53 compared to cells treated with DOX alone (Figure 1B). Additionally, pretreatment with 2 and 5 mM metformin resulted in significant concentration-dependent inhibition of the expression of p53 compared to cells treated with DOX alone (Figure 1C). The same concentrations of metformin, in addition to the 1 mM concentration, significantly inhibited the expression of downstream target p21 compared to cells treated with DOX alone (Figure 1D).

We have recently demonstrated that immortalized EA.hy926 cells and primary HUVECs have a differential response to the senolytic ABT-263 (Abdelgawad et al., 2022a). Therefore, we repeated similar experiments in HUVECs to determine whether metformin has similar effects in both endothelial cell lines. Only higher concentrations of metformin (2 and 5 mM) were used because they were associated with the largest reduction in senescence markers in EA.hy926 cells. Pretreatment of HUVECs with 5 mM metformin significantly inhibited DOX-induced upregulation of p-p53 compared to cells treated with DOX alone (Figure 1E). Similarly, only 5 mM metformin significantly downregulated p53 compared to cells treated with DOX alone (Figure 1F). Both 2 mM and 5 mM metformin significantly decreased the expression of p21 in a concentration-dependent manner compared to cells treated with DOX alone (Figure 1G). Since there is no single specific marker for senescence, the activity of senescence-associated beta-galactosidase (SA-β-gal), which is upregulated in senescent cells, was also evaluated. As shown in Figure 1H, the percentage of SA-β-gal-positive cells significantly increased in HUVECs treated with DOX alone compared to control cells (15.6% vs. 4.1%, respectively). Additionally, DOX-treated cells demonstrated enlarged morphology which is another marker of senescence. Importantly, pretreatment with 5 mM metformin ameliorated the increase in SA-β-gal activity (Figure 1H). However, no statistical difference was observed between DOX-treated HUVECs with and without metformin. Considering that metformin had similar effects in HUVECs and EA.hy926 cells, we chose to use HUVECs as our main endothelial model for subsequent experiments, as they are a more clinically relevant model.

### 3.2 Metformin inhibits DOX-induced secretion of SASP factors and endothelial adhesion molecules in senescent ECs

**FIGURE 2:**
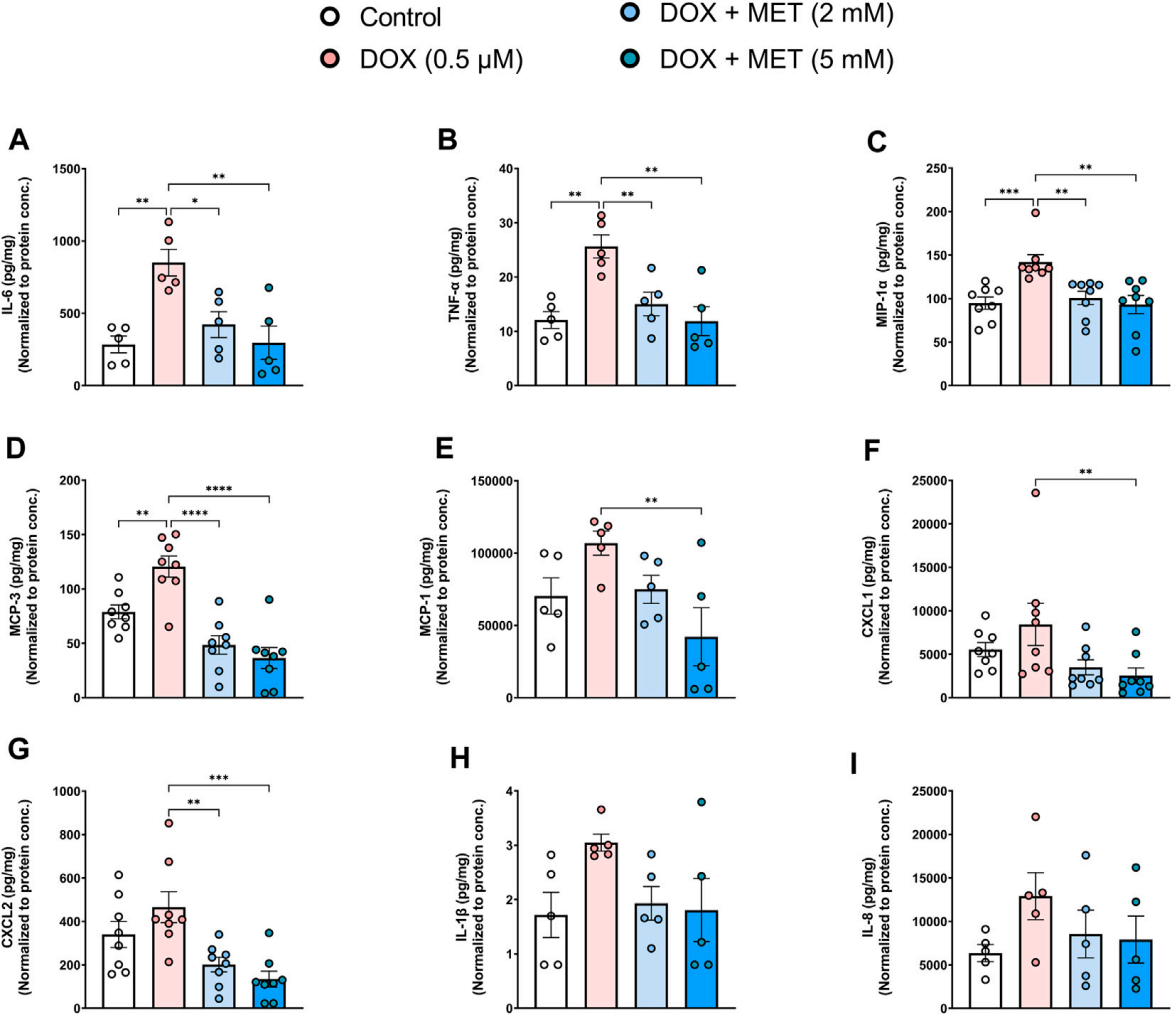
Metformin decreased DOX-induced SASP factors in conditioned media of HUVECs. HUVECs were treated for 24 h with 0.5 µM DOX ± metformin (2 and 5 mM, added 24 h before DOX). Thereafter, DOX was removed and the cells were incubated in DOX-free media with or without metformin for an additional 72 h. Conditioned media were collected and the protein expression of SASP factors including IL-6, TNF-α, MIP-1α, MCP-3, MCP-1, CXCL1, CXCL2, IL-1 β, and IL-8 [**(A–I)**, respectively (n = 5–8)] was determined by Luminex multiplex platform. Values were normalized to the protein concentration of the cells determined by BCA. Values are shown as means ± SEM. Data were analyzed by one-way ANOVA followed by a Dunnet’s multiple comparisons test (Figures 2A,B, D–E, G, I) or non-parametric Kruskal–Wallis tests followed by Dunn’s *post hoc* test (Figures 2C,F,H); * *p* < 0.05, ** *p* < 0.01, *** *p* < 0.001, **** *p* < 0.0001.

Endothelial senescence is also characterized by overexpression of SASP factors which contribute to inflammaging and endothelial dysfunction. We measured the levels of secreted SASP factors in the media of HUVECs following treatment with DOX in the absence or presence of metformin. Treatment of HUVECs with DOX alone induced a significant increase in the concentrations of IL-6 (Figure 2A), TNF-α (Figure 2B), MIP-1α (Figure 2C), and MCP-3 (Figure 2D). The same trend was observed in the expression of other SASP factors including MCP-1, CXCL1, CXCL2, IL-1 β, and IL-8, although the observed increases were not statistically significant (Figures 2E–I). Importantly, pretreatment with 2 mM and 5 mM metformin normalized the levels of cytokines including IL-6 (Figure 2A), TNF-α (Figure 2B), MIP-1α (Figure 2C), MCP-3 (Figure 2D), and CXCL2 (Figure 2G) in a concentration-dependent manner. Treatment with 5 mM metformin, but not 2 mM, significantly reduced the levels of MCP-1 (Figure 2E) and CXCL1 (Figure 2F) in DOX-treated cells. Both IL-1β (Figure 2H) and IL-8 (Figure 2I) were reduced by metformin, although the observed reductions did not reach statistical significance.

**FIGURE 3:**
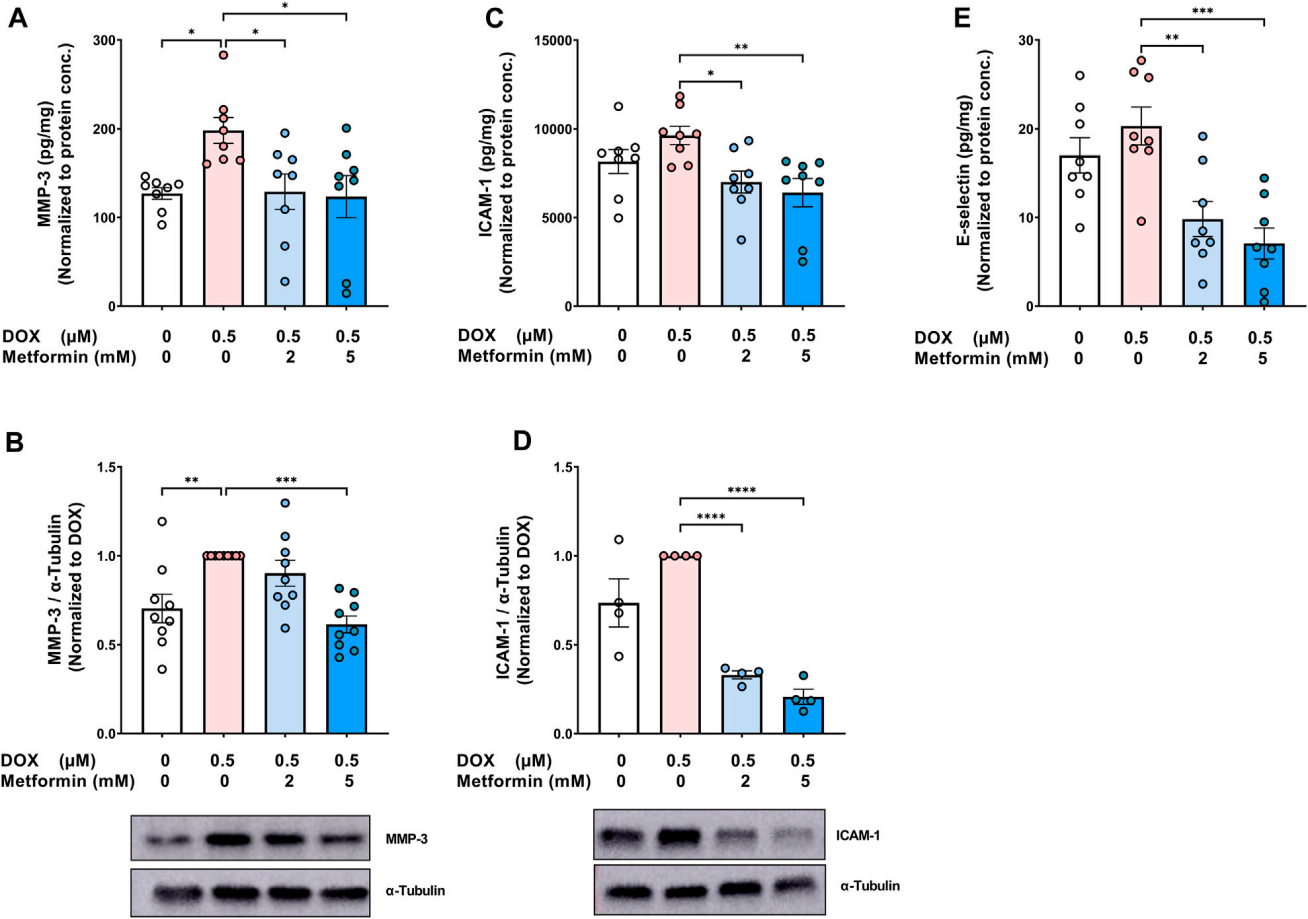
Metformin decreased the protein expression of SASP protease, MMP-3, and endothelial adhesion molecules in HUVECs. HUVECs were treated for 24 h with 0.5 µM DOX ± metformin (2 and 5 mM, added 24 h before DOX). Thereafter, DOX was removed and the cells were incubated in DOX-free media with or without metformin for an additional 72 h. The protein expression of the SASP protease MMP-3 was determined in **(A)** culture media by Luminex and **(B)** cell lysate by western blotting (n = 8–9). Expression of the endothelial adhesion molecules ICAM-1 in **(C)** culture media (n = 8) and **(D)** cell lysate (n = 4), and **(E)** E-selectin (n = 8) in the culture media were determined. Representative images of western blots are shown. Expression values in the media were normalized to the protein concentration of the cells determined by BCA. Values are shown as means ± SEM. Data were analyzed by one-way ANOVA followed by a Dunnet’s multiple comparisons test (Figures 3A,B,D–E) or non-parametric Kruskal–Wallis tests followed by Dunn’s *post hoc* test (Figure 3C); * *p* <0.05, ** *p* <0.01, *** *p* <0.001, **** *p* < 0.0001.

In addition to determining changes in the expression of SASP pro-inflammatory cytokines and chemokines, we assessed the effect of metformin on the SASP protease MMP-3. MMP-3 plays an important role in promoting inflammation, not only in the cardiovascular system, but also in different organs (recently reviewed in (Wan et al., 2021)). DOX alone significantly increased MMP-3 concentration in the culture media (Figure 3A) and its protein expression in cell lysate (Figure 3B). In agreement with the previous SASP results, metformin normalized DOX-induced increase of MMP-3 (Figures 3A,B). Moreover, the expression of endothelial adhesion molecules, including intercellular adhesion molecule-1 (ICAM-1) and E-selectin, was determined since these endothelial markers were previously shown to be highly expressed in senescent HUVECs in models of radiation-induced senescence (Sermsathanasawadi et al., 2009) and replicative senescence (Korybalska et al., 2012). The expression of ICAM-1, in both the culture media (Figure 3C) and cell lysate (Figure 3D), and E-selectin in the culture media (Figure 3E) were increased in DOX-induced senescent cells, although the observed increases were not statistically significant. Metformin significantly decreased the expression of both ICAM-1 and E-selectin compared to cells treated with DOX alone (Figures 3C–E).

### 3.3 Signaling changes associated with the protective effect of metformin against endothelial senescence

**FIGURE 4:**
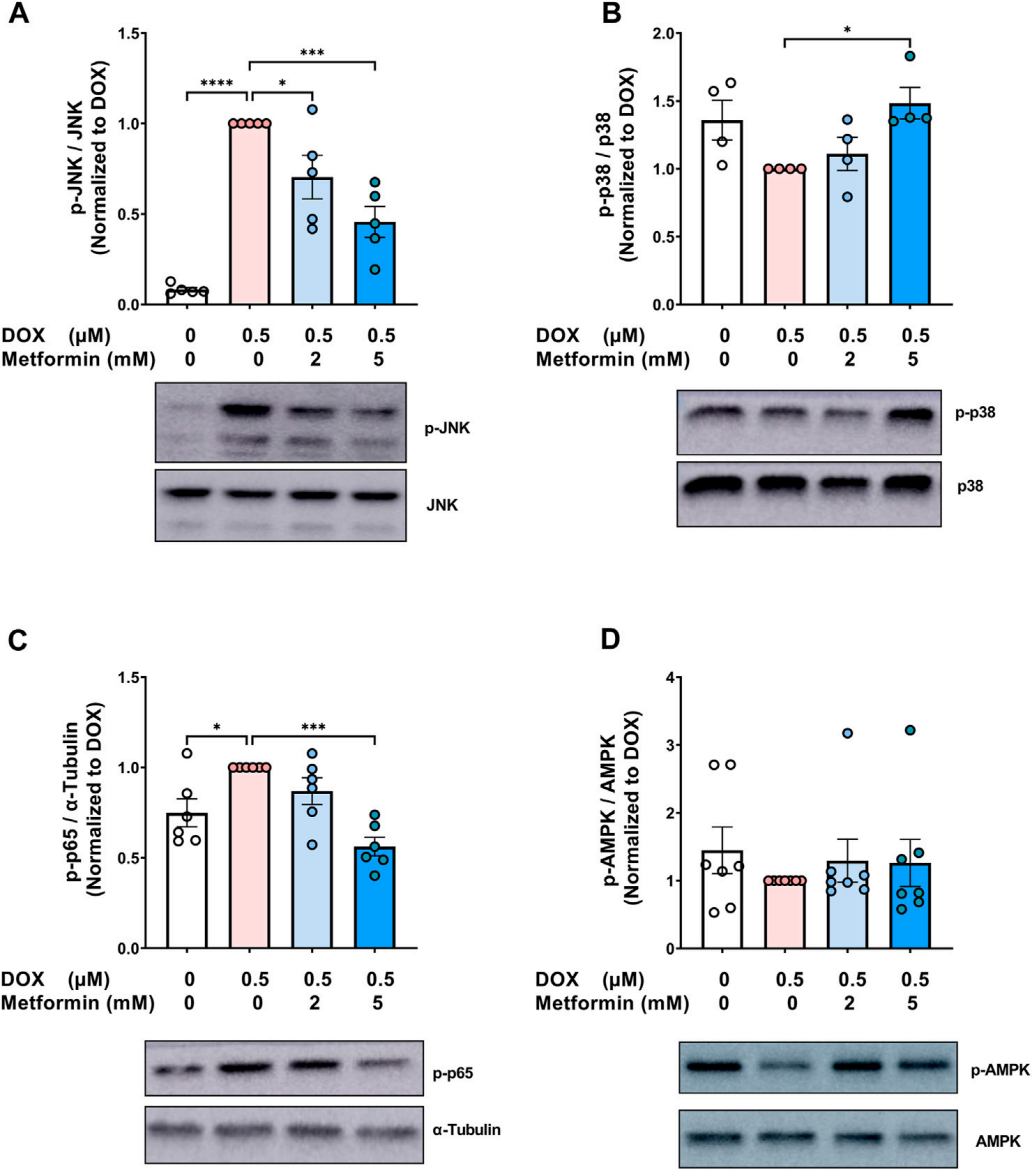
Signaling pathways associated with the protective effect of metformin on endothelial senescence in HUVECs. HUVECs were treated for 24 h with 0.5 µM DOX ± metformin (2 and 5 mM, added 24 h before DOX). Thereafter, DOX was removed and the cells were incubated in DOX-free media with or without metformin for an additional 72 h. Protein expressions of phospho- **(A)** JNK, **(B)** p38, **(C)** NF-κB p65, and **(D)** AMPK (n = 4–7) were quantified using western blot. Representative images of western blots are shown. Values were normalized to total protein or α-tubulin and expressed relative to cells treated with DOX alone. Expressed values are presented as mean ± SEM. Data were analyzed by one-way ANOVA followed by a Dunnet’s multiple comparisons test (Figure 4A,C) or non-parametric Kruskal–Wallis tests followed by Dunn’s *post hoc* test (Figure 4B,D); * *p* <0.05, *** *p* <0.001, **** *p* < 0.0001.

Metformin has pleiotropic properties and can modulate multiple pathways. As a result, many aspects of the mechanisms by which metformin exerts its senomorphic effects are still not fully understood. To gain insights into the mechanistic pathways associated with the modulatory effect of metformin on endothelial senescence, we sought to measure the expression of mitogen-activated protein kinase (MAPK) signaling pathways including c-Jun N-terminal kinase (JNK) and p38 MAPK (p38). MAPKs are activated by stress stimuli, such as the DNA damage response induced by DOX and have been shown to be involved in senescence (Spallarossa et al., 2010; Altieri et al., 2017). JNK was previously demonstrated to be activated in senescent irradiated fibroblasts (Vizioli et al., 2020). Importantly, inhibition of JNK ameliorated the induction of SASP genes without affecting the expression of senescence markers (Vizioli et al., 2020). Our results show that DOX alone resulted in a robust 12-fold increase in JNK phosphorylation (Figure 4A), which is in agreement with previous findings reporting the activation of JNK by DOX in neonatal rat cardiomyocytes (Spallarossa et al., 2009) and endothelial progenitor cells (Spallarossa et al., 2010). Importantly, pretreatment with metformin significantly abrogated DOX-induced JNK activation (Figure 4A). This inhibitory effect of metformin on JNK activation was previously demonstrated in hypoxia/reoxygenation injury model in cardiomyocytes (Hu et al., 2016).

On the other hand, inhibition of p38 was previously reported to suppress both senescence markers and SASP in different models of senescence (Altieri et al., 2017; Huang et al., 2021). In contrast to JNK, no significant changes in the phosphorylation of p38 were observed following treatment with DOX alone (Figure 4B). Surprisingly, 5 mM metformin caused a modest 1.5-fold increase in p38 phosphorylation when compared to DOX alone (Figure 4B). In agreement with our results, no inhibitory effect of metformin was observed on p38 phosphorylation in RAS-induced senescent fibroblasts (Moiseeva et al., 2013), further suggesting that the protective effect of metformin is independent of p38.

The nuclear factor κB (NF-κB) signaling pathway can be activated in response to DNA damage and was shown to be the major inducer of SASP (Salminen et al., 2012). Therefore, it was important to determine the effect of metformin on NF-κB activity. As expected, DOX alone induced NF-κB activation by 1.3-fold compared to control cells, as shown by higher phosphorylation of NF-κB p65 (Figure 4C). Pretreatment with 5 mM metformin abrogated DOX-induced NF-κB activation (Figure 4C). In agreement with this finding, it has been reported that metformin inhibits NF-κB activation in senescent fibroblasts (Moiseeva et al., 2013).

Conflicting results have been reported regarding the involvement of AMPK in the senomorphic effects of metformin. While some studies have reported that metformin inhibits senescence phenotype independently of AMPK (Moiseeva et al., 2013; Park et al., 2022), other studies have reported that AMPK activation is necessary for metformin’s senomorphic and anti-inflammatory effects (Hattori et al., 2006; Chen et al., 2022a). Therefore, we evaluated the effect of DOX ± metformin on AMPK activation. No significant changes were observed in AMPK phosphorylation following DOX treatment with or without metformin (Figure 4D). The lack of AMPK activation by metformin may be attributed to the culture conditions, higher activation of AMPK by metformin was previously shown to be achieved in normoglycemic media than hyperglycemic conditions (Zordoky et al., 2014). Another explanation is that AMPK activation is time-dependent (Zhao et al., 2020). Considering that the ECs were incubated with metformin for 5 days, we speculate that activation may have occurred at an earlier time point. Collectively, our results demonstrated that the protective effects of metformin against DOX-induced endothelial senescence are associated with inhibition of DOX-induced JNK and NF-κB activation.

### 3.4 Metformin protects against LPS-induced hyper-inflammation and exacerbated SASP factors expression in DOX-induced senescent ECs

**FIGURE 5:**
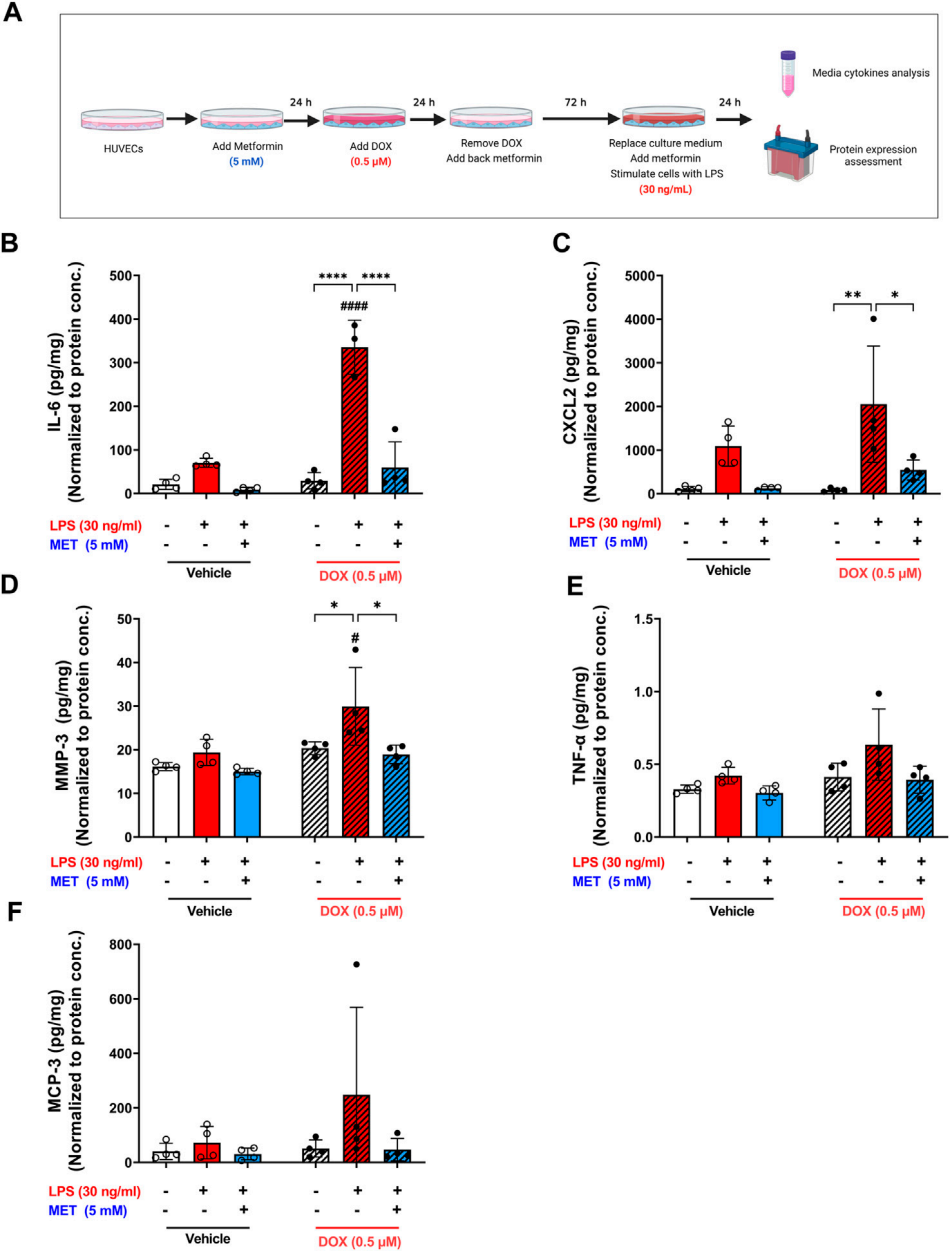
Metformin ameliorated LPS-triggered hyper-inflammation in DOX-induced senescent HUVECs. **(A)** Schematic diagram of the experimental design. HUVECs were treated for 24 h with 0.5 µM DOX ±5 mM metformin (added 24 h before DOX) or left untreated. Thereafter, DOX was removed and the cells were incubated in DOX-free media with or without metformin for an additional 72 h. The media were changed so that the assessed SASP factors in the media reflect only the effect of LPS. Then, cells were stimulated with LPS (30 ng/mL) for an additional 24 h. Thereafter, conditioned media were collected and the protein expression of SASP factors including **(B)** IL-6, **(C)** CXCL2, **(D)** MMP-3, **(E)** TNF-α, and **(F)** MCP-3 was determined by Luminex (n = 4). Values were normalized to the protein concentration of the cells determined by BCA. Expressed values are presented as mean ± SEM. Data were analyzed by two-way ANOVA followed by a Tukey multiple comparisons test. * compared to different treatment within the same group; * *p* <0.05, ** *p* <0.01, **** *p* < 0.0001. # compared to non-senescent cells with the same treatment; # *p* <0.05, #### *p* < 0.0001.

Lipopolysaccharide (LPS) is a bacterial toxin found on the outer membrane of gram-negative bacteria. When LPS enters the body, it can stimulate the immune system and cause a severe, systemic inflammatory response. Notably, ECs express specific receptors such as toll-like receptor 4 (TLR4) that interact with LPS, making them susceptible to LPS-induced inflammation. Recent studies demonstrate that senescent ECs are even more vulnerable to LPS-induced inflammation compared to non-senescent cells (Suzuki et al., 2019; Budamagunta et al., 2021; Camell et al., 2021). Importantly, these studies used other models of senescence than DOX-induced senescence. Therefore, we sought to characterize the effect of LPS-induced inflammation in DOX-induced senescent HUVECs. We exposed non-senescent and DOX-induced senescent HUVECs to LPS for 24 h following establishment of senescence and removing the culture medium that contain already-secreted SASP factors as illustrated in Figure 5A. A significant hyper-stimulatory response to LPS was observed in senescent HUVECs compared to non-senescent cells, demonstrated by remarkable increase of the secretion of SASP factors in the media including IL-6, CXCL2, and MMP3 (Figures 5B–D). Importantly, 5 mM metformin significantly inhibited LPS-induced hyper-inflammation and almost normalized the level of these cytokines in the media. Other SASP factors including TNF-α (Figure 5E) and MCP-3 (Figure 5F) demonstrated the same trend; however, they were not significant.

**FIGURE 6:**
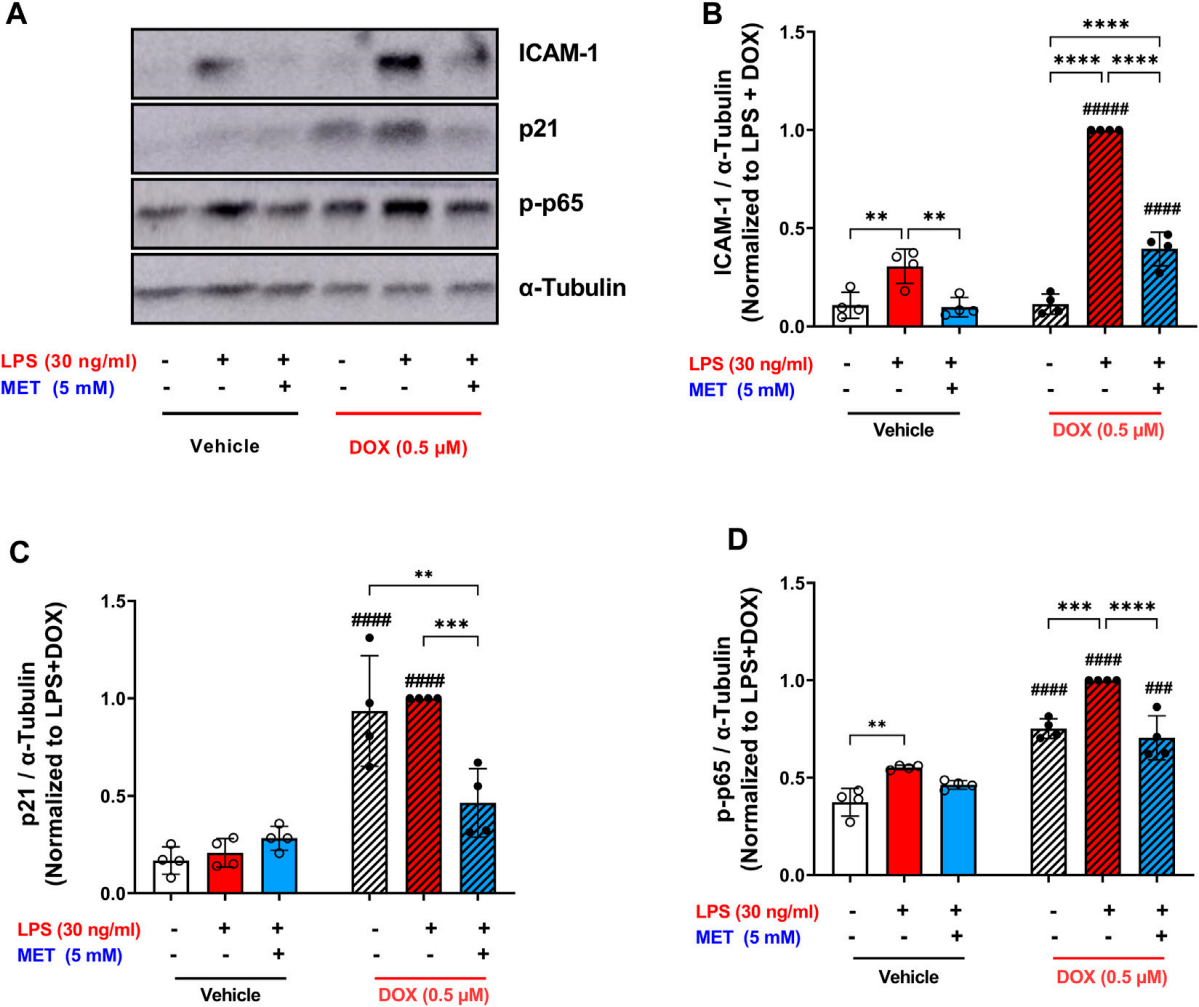
Metformin suppressed the expression of ICAM-1 and prevented NF-κB activation following LPS stimulation. HUVECs were treated for 24 h with 0.5 µM DOX ± 5 mM metformin (added 24 h before DOX) or left untreated. Thereafter DOX was removed and the cells were incubated in DOX-free media with or without metformin for an additional 72 h. Then, cells were stimulated with LPS (30 ng/mL) for an additional 24 h. **(A)** Representative images of western blot are shown and the expression levels of **(B)** ICAM-1, **(C)** p21, and **(D)** phospho-NF-κB p65 were measured (n = 4). Values were normalized to α-tubulin and expressed relative to DOX + LPS treated cells. Expressed values are presented as mean ± SEM. Data were analyzed by two-way ANOVA followed by a Tukey multiple comparisons test. * compared to different treatment within the same group; ** *p* <0.01, *** *p* <0.001, **** *p* < 0.0001. # compared to non-senescent cells with the same treatment; # *p* <0.05, ### *p* <0.001, #### *p* < 0.0001.

The same trend was observed in the protein expression of the adhesion molecule ICAM-1. LPS triggered a 9-fold upregulation in the expression of ICAM-1 in DOX-induced senescent HUVECs compared to 2.8-fold increase in non-senescent HUVECs (Figures 6A,B). Metformin significantly reversed this upregulation (Figure 6B).

To confirm the senescence phenotype, the expression of p21 was measured as a surrogate marker of senescence. Consistent with our previous results (Figure 1G), cells treated with DOX alone demonstrated higher expression of p21 which was maintained after LPS stimulation (Figure 6C). Pretreatment with metformin significantly downregulated the expression of p21 (Figure 6C). Mechanistically, exposure to LPS increased the phosphorylation of NF-κB p65 both in senescent and non-senescent HUVECs (Figure 6D). Metformin significantly inhibited NF-κB activation as shown by decreased phosphorylation of NF-κB p65 (Figure 6D). Together these findings suggest that metformin maintains its senomorphic properties even in the presence of hyper-inflammation in senescent ECs. Of note, since metformin was added before DOX and throughout all steps, these observed effects may either be due to its senomorphic effects against DOX-induced senescence, direct anti-inflammatory effects against LPS, or a combination of both.

## 4 Discussion

Doxorubicin (DOX) is a widely used chemotherapy drug that has been in use since the 1960s. Despite its effectiveness, DOX’s clinical utility is limited due to its adverse cardiovascular effects. Others and we have previously demonstrated that DOX induces senescence in endothelial cells (ECs) (Yang et al., 2015; Chen et al., 2021a; Matacchione et al., 2021; Misuth et al., 2021; Abdelgawad et al., 2022a), which may contribute to the deleterious outcomes associated with DOX. ECs play several important roles such as maintaining vascular tone, initiating angiogenesis, and acting as a barrier to molecules circulating in the blood, all of which are impaired when ECs become senescent (Abdelgawad et al., 2022b). One characteristic feature of senescent cells is the secretion of SASP, which consists of multiple components including pro-inflammatory cytokines, chemokines, and proteases (Coppe et al., 2008). A recent study showed that senescent ECs have higher levels of SASP expression compared to other senescent cell types (Schafer et al., 2020). Importantly, the accumulation of SASP can have deleterious effects on the cardiovascular system. Of note, these deleterious effects extend beyond the cardiovascular system, as overexpression of SASP has been shown to promote cancer progression (Hwang et al., 2020) and trigger a hyper-inflammatory response to inflammatory stimuli such as infections (Budamagunta et al., 2021; Camell et al., 2021).

Targeting senescent ECs could be a promising strategy to mitigate the complications of DOX-induced endothelial senescence. Recent findings have demonstrated that the removal of senescent cells using a genetic approach following DOX administration in mice restored endothelium-dependent dilation, suggesting the important role of senescence in mediating DOX-induced vascular dysfunction (Hutton et al., 2021). Others and we have shown that pharmacological approaches such as senolytics can selectively induce apoptosis in ECs (Zhu et al., 2017; Abdelgawad et al., 2022a). However, senolytics have limitations, including the potential to kill non-senescent cells and the risk of some senolytics to trigger thrombocytopenia (Leverson et al., 2015; Hwang et al., 2018). As a result, senomorphics may be another pharmacological alternative because they modulate the senescence phenotype by downregulating the detrimental effects of SASP without eliminating senescent cells.

Accumulating evidence has previously shown metformin to demonstrate senomorphic and anti-aging actions in different models of senescence including radiation (Hansel et al., 2021; Park et al., 2022), hyperglycemia (Arunachalam et al., 2014), and oxidative stress-induced senescence (Chen et al., 2021b). Two clinical trials, including MILES (Metformin In Longevity Study), and TAME (Targeting Aging with Metformin), have been designed to further identify the anti-aging effects of metformin (Barzilai et al., 2016; Kulkarni et al., 2018). However, the effect of metformin on DOX-induced endothelial senescence has not been established. Recent evidence suggests that the signature of senescence displayed by cells can vary greatly depending on the cell type and the inducer of senescence (Casella et al., 2019; Abdelgawad et al., 2022b). Moreover, we have recently shown that the effect of senolytics can also differ, as demonstrated by the differential response to ABT-263 in different senescent endothelial cell lines (Abdelgawad et al., 2022a). Therefore, we evaluated for the first time the effect of metformin on modulating DOX-induced senescence in ECs. Our results demonstrate that metformin abrogates DOX-induced endothelial senescence, as evidenced by the suppressed expression of senescence markers and decreased SA-β-gal activity. These findings were validated using two different endothelial cell lines: primary HUVECs and immortalized EA.hy926 endothelial-derived cells, and demonstrated that metformin suppressed DOX-induced senescence markers to the same extent in both cell lines.

The present study demonstrates for the first time that treatment of DOX-induced senescent ECs with metformin inhibits the secretion of SASP factors including pro-inflammatory cytokines (IL-6, TNF-α), chemokines (CXCL1, CXCL2, MCP 1 and 3, and MIP-1α), and proteases (MMP-3). Excessive secretion of SASP promotes vascular inflammation and can contribute to DOX-induced cardiovascular complications. Venturini et al. (2020) recently demonstrated through an *in vitro* model that SASP factors released due to DOX-induced endothelial senescence stimulate platelet activation and aggregation, which can contribute to atherothrombotic events. In agreement with the observed anti-inflammatory effect, metformin was previously shown to suppress the expression of SASP factors in RAS-induced senescent fibroblasts (Moiseeva et al., 2013) and angiotensin-II-induced senescent vascular smooth muscle cells (Tai et al., 2022). Moreover, a recent clinical study demonstrated that treatment with metformin was associated with a lower expression of SASP factors (IL-6 and TNF-α) in B cells isolated from elderly population (Frasca et al., 2021). Our findings also showed that metformin markedly decreased the expression of adhesion molecules ICAM-1 and E-selectin in DOX-induced senescent ECs. Higher expression of adhesion molecules can increase the risk of vascular complications by facilitating the recruitment and aggregation of leukocytes on the endothelial surface, which triggers vascular inflammation. Together, these findings suggest that metformin can be a promising senomorphic approach to protect against DOX-induced vascular aging and vascular inflammation.

Recent evidence suggests that the effects of SASP can go beyond the cardiovascular system. Indeed, SASP-induced inflammaging was recently shown to induce a hyper-inflammatory response upon exposure to further inflammatory insults, such as the cytokine storm induced by COVID-19 (Camell et al., 2021). The same study showed that treating aged mice with the pathogen-associated molecular pattern factor LPS increased the serum levels of inflammatory SASP factors compared to young mice (Camell et al., 2021). The same concept was demonstrated *in vitro*, where the stimulation of radiation-induced senescent ECs with LPS resulted in a higher induction of inflammation compared to non-senescent cells (Budamagunta et al., 2021; Camell et al., 2021). Together, these results suggest that the higher vulnerability of the aged population to COVID-19 related mortality may be attributed, at least in part, to the accumulation of senescent cells and the resulting hyper-inflammatory response. The same explanation may also be applicable to other conditions with a high burden of senescence, such as cancer survivors. In agreement with this hypothesis, a recent retrospective observational study demonstrated that childhood cancer survivors are at a higher risk of developing severe infections that require hospitalization (Chehab et al., 2022). Furthermore, a recent population-based study in Italy showed that cancer survivors are at the same risk of infection with COVID-19, but have an increased risk of mortality once infected (Mangone et al., 2021). The current study showed that LPS induced a hyper-inflammatory response in DOX-induced senescent ECs compared to non-senescent cells. To our knowledge, this is the first study to report the effect of LPS on DOX-induced senescent cells. These results can provide a mechanistic explanation for the clinical findings in cancer survivors.

Consequently, decreasing the burden of senescence can be a promising strategy for mitigating the severity of bacterial infections in these vulnerable populations. Recently, senolytics were demonstrated to decrease the mortality of aged mice exposed to a mouse β-coronavirus, further supporting the detrimental role of senescence in infections (Camell et al., 2021). In the current work, we showed that metformin significantly abolished LPS-induced hyper-inflammation and normalizes the level of SASP factors in DOX-induced senescent ECs. The demonstrated anti-inflammatory effects support, at least in part, recent data from the COVID-OUT trial demonstrating a 42% relative decrease in the incidence of Long Covid in patients treated with metformin (Bramante et al., 2022). Moreover, a recent review highlighted the anti-inflammatory effect of metformin on the microvasculature as a major contributor to better outcomes in COVID-19 patients (Wiernsperger et al., 2022).

In conclusion, the current study showed that metformin protected against DOX-induced endothelial senescence as evidenced by the abrogation of senescence markers and downregulation of SASP factors and adhesion molecules. This protective effect was associated with inhibition of JNK and NF-κB pathways. Additionally, we showed that DOX-induced senescent ECs exhibited a hyper-inflammatory response to LPS compared to non-senescent cells. Importantly, metformin ameliorated this hyper-inflammation. Together, these findings suggest that metformin may serve as a promising drug for mitigating DOX-induced endothelial senescence and the resulting complications.

## Data Availability

The raw data supporting the conclusions of this article will be made available by the authors, without undue reservation.
