# Supplementary material for: Metformin mitigates SASP secretion and LPS-triggered hyper-inflammation in Doxorubicin-induced senescent endothelial cells
Source: Front Aging. 2023 Apr 24;4:1170434. doi: 10.3389/fragi.2023.1170434 (PMC10164964; doi:10.3389/fragi.2023.1170434)
Supplement: Supplementary file 1 [file Table1.DOCX]

Supplementary Material

Metformin Mitigates SASP Secretion and LPS-triggered Hyper-inflammation in Doxorubicin-induced Senescent Endothelial Cells

**Ibrahim Y. Abdelgawad^†^, Kevin Agostinucci^†^, Bushra Sadaf, Marianne K. O. Grant, and Beshay N. Zordoky***

*** Correspondence:** Beshay Zordoky:zordo001@umn.edu

# Supplementary Data

Uncropped western blot images.
