# Supplementary material for: Metformin mitigates SASP secretion and LPS-triggered hyper-inflammation in Doxorubicin-induced senescent endothelial cells
Source: Front Aging. 2023 Apr 24;4:1170434. doi: 10.3389/fragi.2023.1170434 (PMC10164964; doi:10.3389/fragi.2023.1170434)
Supplement: Supplementary file 2 [file Presentation1.PPTX]

## Slide 1
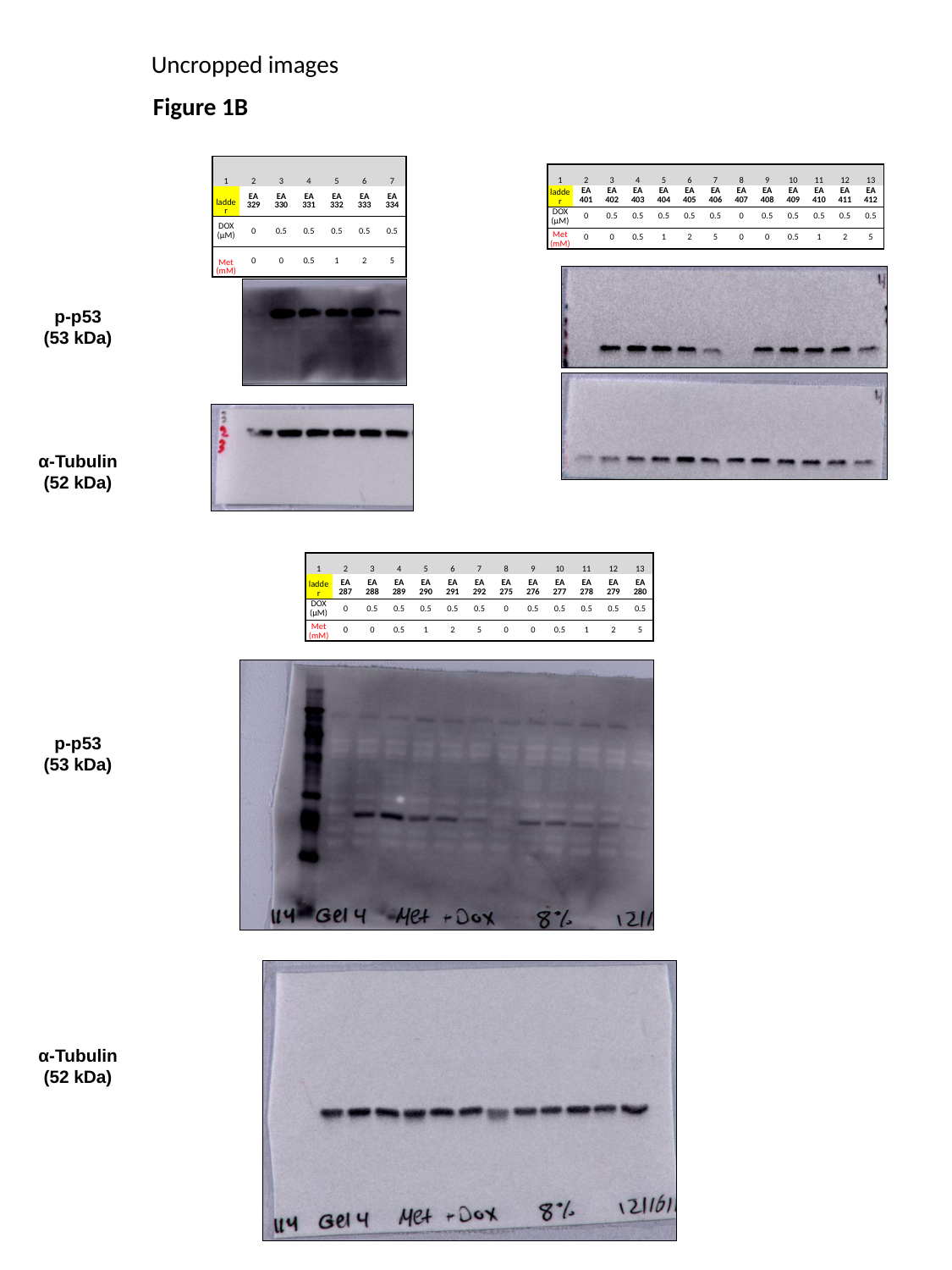

Uncropped images
Figure 1B
| 1 | 2 | 3 | 4 | 5 | 6 | 7 |
| --- | --- | --- | --- | --- | --- | --- |
| ladder | EA 329 | EA 330 | EA 331 | EA 332 | EA 333 | EA 334 |
| DOX (µM) | 0 | 0.5 | 0.5 | 0.5 | 0.5 | 0.5 |
| Met (mM) | 0 | 0 | 0.5 | 1 | 2 | 5 |
| 1 | 2 | 3 | 4 | 5 | 6 | 7 | 8 | 9 | 10 | 11 | 12 | 13 |
| --- | --- | --- | --- | --- | --- | --- | --- | --- | --- | --- | --- | --- |
| ladder | EA 401 | EA 402 | EA 403 | EA 404 | EA 405 | EA 406 | EA 407 | EA 408 | EA 409 | EA 410 | EA 411 | EA 412 |
| DOX (µM) | 0 | 0.5 | 0.5 | 0.5 | 0.5 | 0.5 | 0 | 0.5 | 0.5 | 0.5 | 0.5 | 0.5 |
| Met (mM) | 0 | 0 | 0.5 | 1 | 2 | 5 | 0 | 0 | 0.5 | 1 | 2 | 5 |
p-p53
(53 kDa)
α-Tubulin
(52 kDa)
| 1 | 2 | 3 | 4 | 5 | 6 | 7 | 8 | 9 | 10 | 11 | 12 | 13 |
| --- | --- | --- | --- | --- | --- | --- | --- | --- | --- | --- | --- | --- |
| ladder | EA 287 | EA 288 | EA 289 | EA 290 | EA 291 | EA 292 | EA 275 | EA 276 | EA 277 | EA 278 | EA 279 | EA 280 |
| DOX (µM) | 0 | 0.5 | 0.5 | 0.5 | 0.5 | 0.5 | 0 | 0.5 | 0.5 | 0.5 | 0.5 | 0.5 |
| Met (mM) | 0 | 0 | 0.5 | 1 | 2 | 5 | 0 | 0 | 0.5 | 1 | 2 | 5 |
p-p53
(53 kDa)
α-Tubulin
(52 kDa)

## Slide 2
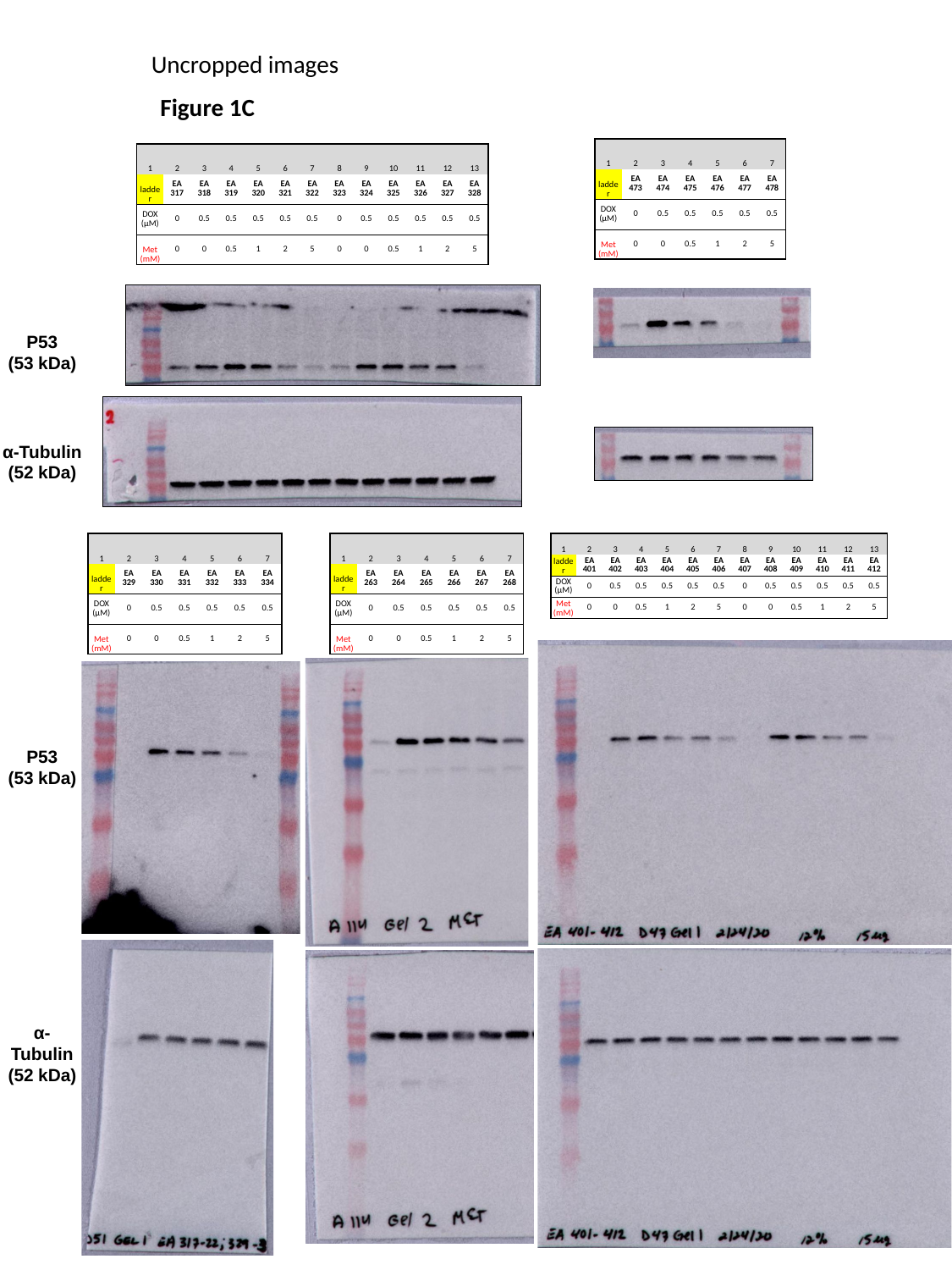

Uncropped images
Figure 1C
| 1 | 2 | 3 | 4 | 5 | 6 | 7 |
| --- | --- | --- | --- | --- | --- | --- |
| ladder | EA 473 | EA 474 | EA 475 | EA 476 | EA 477 | EA 478 |
| DOX (µM) | 0 | 0.5 | 0.5 | 0.5 | 0.5 | 0.5 |
| Met (mM) | 0 | 0 | 0.5 | 1 | 2 | 5 |
| 1 | 2 | 3 | 4 | 5 | 6 | 7 | 8 | 9 | 10 | 11 | 12 | 13 |
| --- | --- | --- | --- | --- | --- | --- | --- | --- | --- | --- | --- | --- |
| ladder | EA 317 | EA 318 | EA 319 | EA 320 | EA 321 | EA 322 | EA 323 | EA 324 | EA 325 | EA 326 | EA 327 | EA 328 |
| DOX (µM) | 0 | 0.5 | 0.5 | 0.5 | 0.5 | 0.5 | 0 | 0.5 | 0.5 | 0.5 | 0.5 | 0.5 |
| Met (mM) | 0 | 0 | 0.5 | 1 | 2 | 5 | 0 | 0 | 0.5 | 1 | 2 | 5 |
P53
(53 kDa)
α-Tubulin
(52 kDa)
| 1 | 2 | 3 | 4 | 5 | 6 | 7 |
| --- | --- | --- | --- | --- | --- | --- |
| ladder | EA 329 | EA 330 | EA 331 | EA 332 | EA 333 | EA 334 |
| DOX (µM) | 0 | 0.5 | 0.5 | 0.5 | 0.5 | 0.5 |
| Met (mM) | 0 | 0 | 0.5 | 1 | 2 | 5 |
| 1 | 2 | 3 | 4 | 5 | 6 | 7 |
| --- | --- | --- | --- | --- | --- | --- |
| ladder | EA 263 | EA 264 | EA 265 | EA 266 | EA 267 | EA 268 |
| DOX (µM) | 0 | 0.5 | 0.5 | 0.5 | 0.5 | 0.5 |
| Met (mM) | 0 | 0 | 0.5 | 1 | 2 | 5 |
| 1 | 2 | 3 | 4 | 5 | 6 | 7 | 8 | 9 | 10 | 11 | 12 | 13 |
| --- | --- | --- | --- | --- | --- | --- | --- | --- | --- | --- | --- | --- |
| ladder | EA 401 | EA 402 | EA 403 | EA 404 | EA 405 | EA 406 | EA 407 | EA 408 | EA 409 | EA 410 | EA 411 | EA 412 |
| DOX (µM) | 0 | 0.5 | 0.5 | 0.5 | 0.5 | 0.5 | 0 | 0.5 | 0.5 | 0.5 | 0.5 | 0.5 |
| Met (mM) | 0 | 0 | 0.5 | 1 | 2 | 5 | 0 | 0 | 0.5 | 1 | 2 | 5 |
P53
(53 kDa)
α-
Tubulin
(52 kDa)

## Slide 3
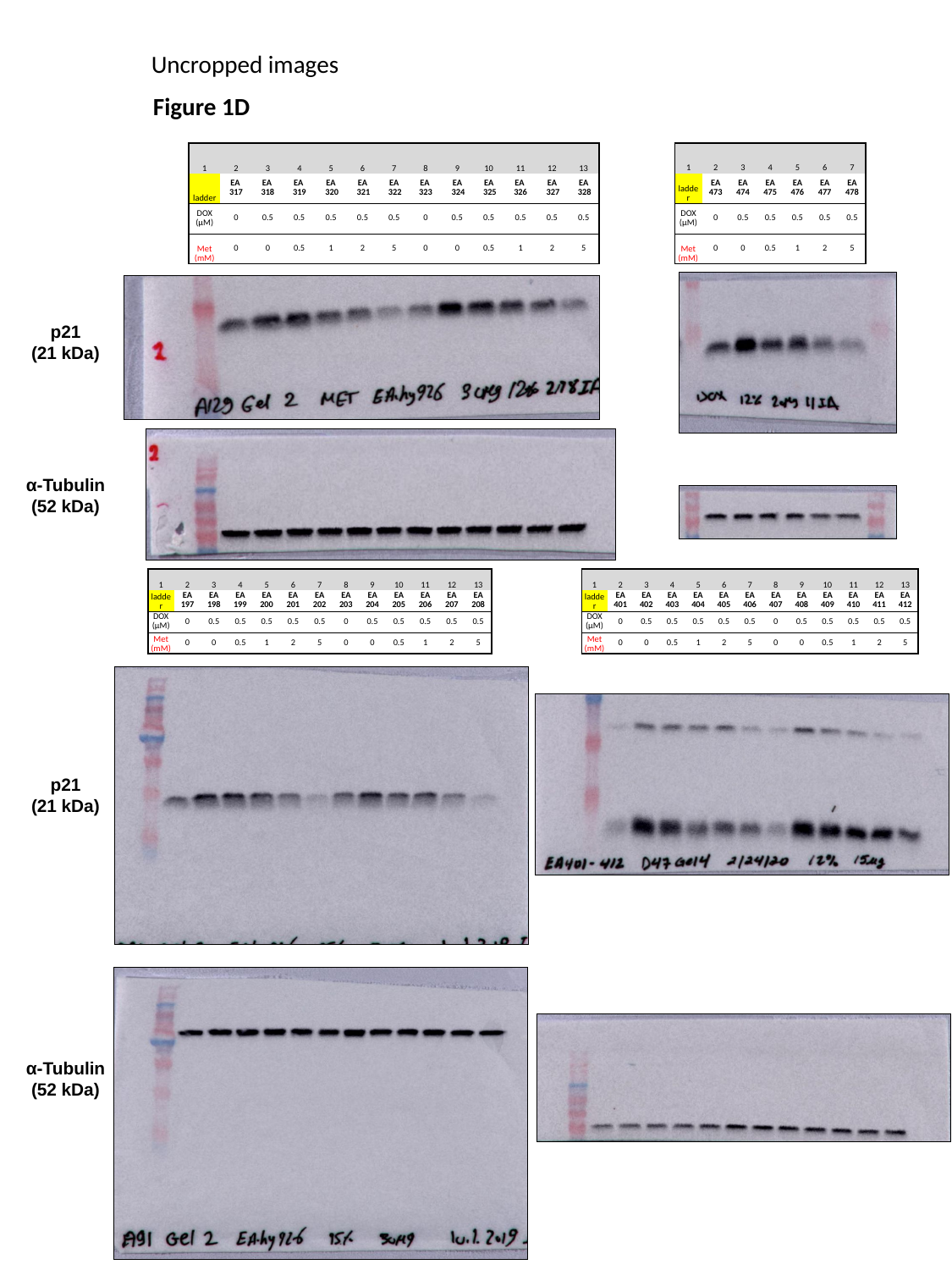

Uncropped images
Figure 1D
| 1 | 2 | 3 | 4 | 5 | 6 | 7 |
| --- | --- | --- | --- | --- | --- | --- |
| ladder | EA 473 | EA 474 | EA 475 | EA 476 | EA 477 | EA 478 |
| DOX (µM) | 0 | 0.5 | 0.5 | 0.5 | 0.5 | 0.5 |
| Met (mM) | 0 | 0 | 0.5 | 1 | 2 | 5 |
| 1 | 2 | 3 | 4 | 5 | 6 | 7 | 8 | 9 | 10 | 11 | 12 | 13 |
| --- | --- | --- | --- | --- | --- | --- | --- | --- | --- | --- | --- | --- |
| ladder | EA 317 | EA 318 | EA 319 | EA 320 | EA 321 | EA 322 | EA 323 | EA 324 | EA 325 | EA 326 | EA 327 | EA 328 |
| DOX (µM) | 0 | 0.5 | 0.5 | 0.5 | 0.5 | 0.5 | 0 | 0.5 | 0.5 | 0.5 | 0.5 | 0.5 |
| Met (mM) | 0 | 0 | 0.5 | 1 | 2 | 5 | 0 | 0 | 0.5 | 1 | 2 | 5 |
p21
(21 kDa)
α-Tubulin
(52 kDa)
| 1 | 2 | 3 | 4 | 5 | 6 | 7 | 8 | 9 | 10 | 11 | 12 | 13 |
| --- | --- | --- | --- | --- | --- | --- | --- | --- | --- | --- | --- | --- |
| ladder | EA 197 | EA 198 | EA 199 | EA 200 | EA 201 | EA 202 | EA 203 | EA 204 | EA 205 | EA 206 | EA 207 | EA 208 |
| DOX (µM) | 0 | 0.5 | 0.5 | 0.5 | 0.5 | 0.5 | 0 | 0.5 | 0.5 | 0.5 | 0.5 | 0.5 |
| Met (mM) | 0 | 0 | 0.5 | 1 | 2 | 5 | 0 | 0 | 0.5 | 1 | 2 | 5 |
| 1 | 2 | 3 | 4 | 5 | 6 | 7 | 8 | 9 | 10 | 11 | 12 | 13 |
| --- | --- | --- | --- | --- | --- | --- | --- | --- | --- | --- | --- | --- |
| ladder | EA 401 | EA 402 | EA 403 | EA 404 | EA 405 | EA 406 | EA 407 | EA 408 | EA 409 | EA 410 | EA 411 | EA 412 |
| DOX (µM) | 0 | 0.5 | 0.5 | 0.5 | 0.5 | 0.5 | 0 | 0.5 | 0.5 | 0.5 | 0.5 | 0.5 |
| Met (mM) | 0 | 0 | 0.5 | 1 | 2 | 5 | 0 | 0 | 0.5 | 1 | 2 | 5 |
p21
(21 kDa)
α-Tubulin
(52 kDa)

## Slide 4
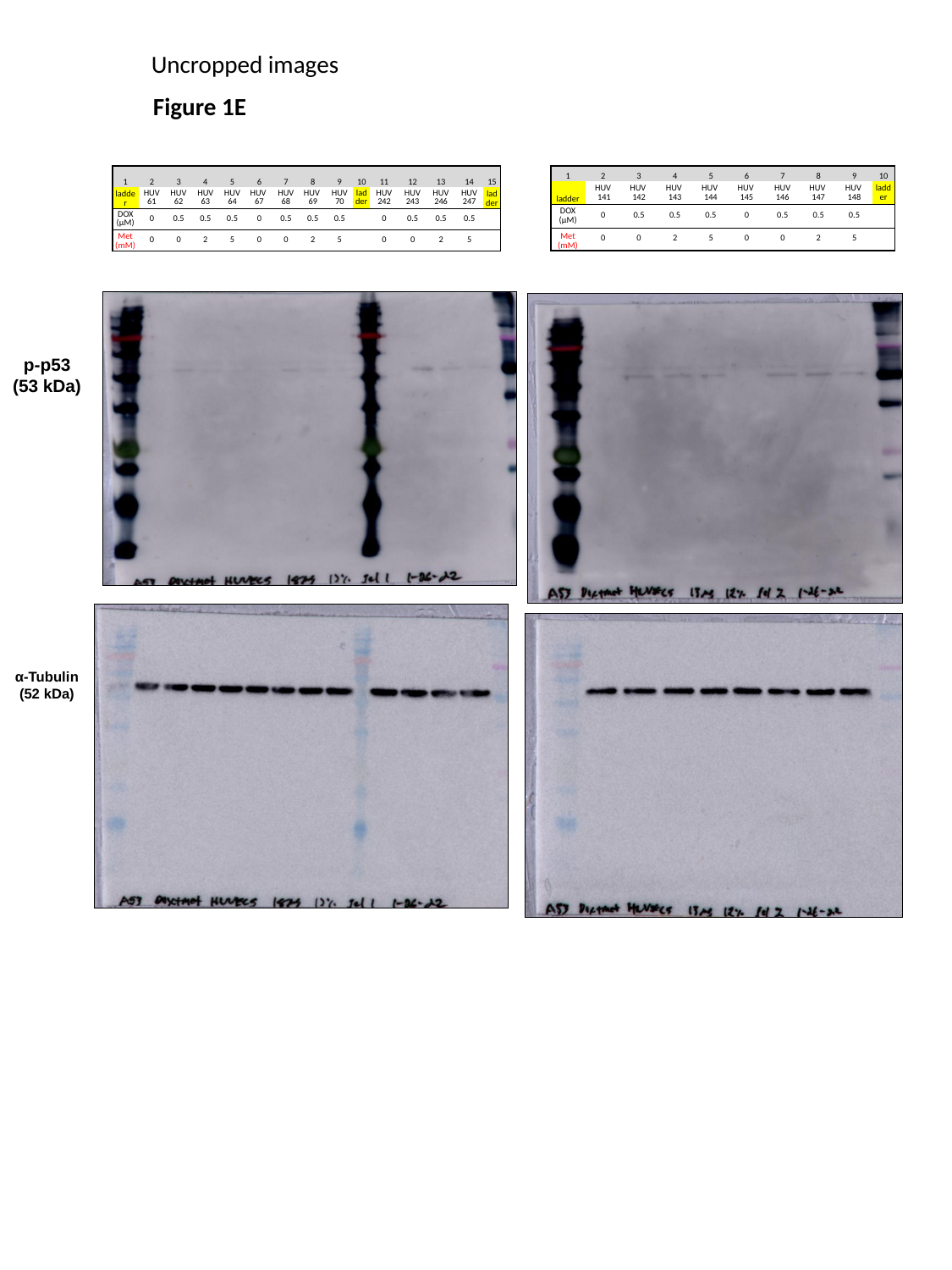

Uncropped images
Figure 1E
| 1 | 2 | 3 | 4 | 5 | 6 | 7 | 8 | 9 | 10 | 11 | 12 | 13 | 14 | 15 |
| --- | --- | --- | --- | --- | --- | --- | --- | --- | --- | --- | --- | --- | --- | --- |
| ladder | HUV 61 | HUV 62 | HUV 63 | HUV 64 | HUV 67 | HUV 68 | HUV 69 | HUV 70 | ladder | HUV 242 | HUV 243 | HUV 246 | HUV 247 | ladder |
| DOX (µM) | 0 | 0.5 | 0.5 | 0.5 | 0 | 0.5 | 0.5 | 0.5 | | 0 | 0.5 | 0.5 | 0.5 | |
| Met (mM) | 0 | 0 | 2 | 5 | 0 | 0 | 2 | 5 | | 0 | 0 | 2 | 5 | |
| 1 | 2 | 3 | 4 | 5 | 6 | 7 | 8 | 9 | 10 |
| --- | --- | --- | --- | --- | --- | --- | --- | --- | --- |
| ladder | HUV 141 | HUV 142 | HUV 143 | HUV 144 | HUV 145 | HUV 146 | HUV 147 | HUV 148 | ladder |
| DOX (µM) | 0 | 0.5 | 0.5 | 0.5 | 0 | 0.5 | 0.5 | 0.5 | |
| Met (mM) | 0 | 0 | 2 | 5 | 0 | 0 | 2 | 5 | |
p-p53
(53 kDa)
α-Tubulin
(52 kDa)

## Slide 5
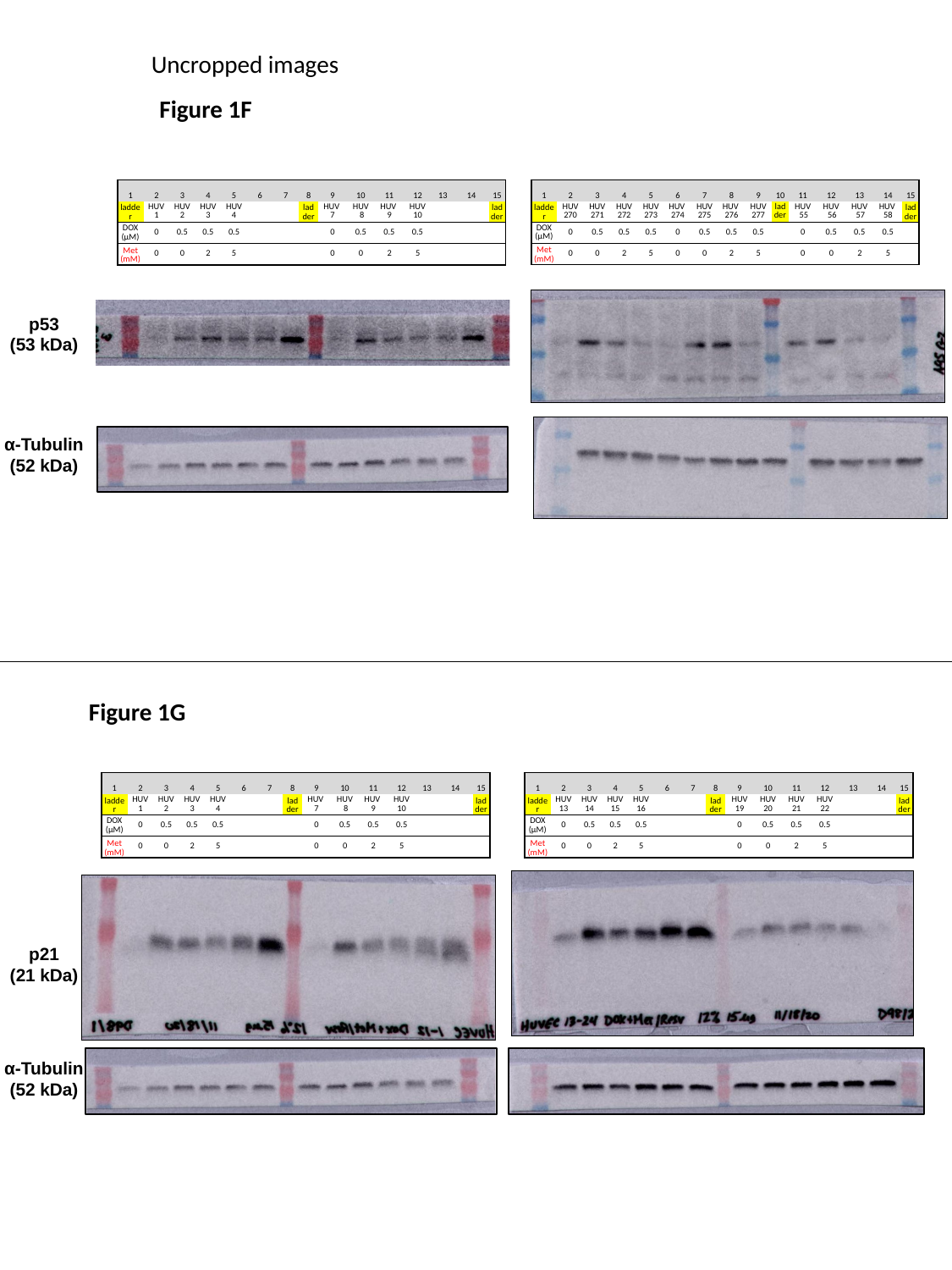

Uncropped images
Figure 1F
| 1 | 2 | 3 | 4 | 5 | 6 | 7 | 8 | 9 | 10 | 11 | 12 | 13 | 14 | 15 |
| --- | --- | --- | --- | --- | --- | --- | --- | --- | --- | --- | --- | --- | --- | --- |
| ladder | HUV 1 | HUV 2 | HUV 3 | HUV 4 | | | ladder | HUV 7 | HUV 8 | HUV 9 | HUV 10 | | | ladder |
| DOX (µM) | 0 | 0.5 | 0.5 | 0.5 | | | | 0 | 0.5 | 0.5 | 0.5 | | | |
| Met (mM) | 0 | 0 | 2 | 5 | | | | 0 | 0 | 2 | 5 | | | |
| 1 | 2 | 3 | 4 | 5 | 6 | 7 | 8 | 9 | 10 | 11 | 12 | 13 | 14 | 15 |
| --- | --- | --- | --- | --- | --- | --- | --- | --- | --- | --- | --- | --- | --- | --- |
| ladder | HUV 270 | HUV 271 | HUV 272 | HUV 273 | HUV 274 | HUV 275 | HUV 276 | HUV 277 | ladder | HUV 55 | HUV 56 | HUV 57 | HUV 58 | ladder |
| DOX (µM) | 0 | 0.5 | 0.5 | 0.5 | 0 | 0.5 | 0.5 | 0.5 | | 0 | 0.5 | 0.5 | 0.5 | |
| Met (mM) | 0 | 0 | 2 | 5 | 0 | 0 | 2 | 5 | | 0 | 0 | 2 | 5 | |
p53
(53 kDa)
α-Tubulin
(52 kDa)
Figure 1G
| 1 | 2 | 3 | 4 | 5 | 6 | 7 | 8 | 9 | 10 | 11 | 12 | 13 | 14 | 15 |
| --- | --- | --- | --- | --- | --- | --- | --- | --- | --- | --- | --- | --- | --- | --- |
| ladder | HUV 1 | HUV 2 | HUV 3 | HUV 4 | | | ladder | HUV 7 | HUV 8 | HUV 9 | HUV 10 | | | ladder |
| DOX (µM) | 0 | 0.5 | 0.5 | 0.5 | | | | 0 | 0.5 | 0.5 | 0.5 | | | |
| Met (mM) | 0 | 0 | 2 | 5 | | | | 0 | 0 | 2 | 5 | | | |
| 1 | 2 | 3 | 4 | 5 | 6 | 7 | 8 | 9 | 10 | 11 | 12 | 13 | 14 | 15 |
| --- | --- | --- | --- | --- | --- | --- | --- | --- | --- | --- | --- | --- | --- | --- |
| ladder | HUV 13 | HUV 14 | HUV 15 | HUV 16 | | | ladder | HUV 19 | HUV 20 | HUV 21 | HUV 22 | | | ladder |
| DOX (µM) | 0 | 0.5 | 0.5 | 0.5 | | | | 0 | 0.5 | 0.5 | 0.5 | | | |
| Met (mM) | 0 | 0 | 2 | 5 | | | | 0 | 0 | 2 | 5 | | | |
p21
(21 kDa)
α-Tubulin
(52 kDa)

## Slide 6
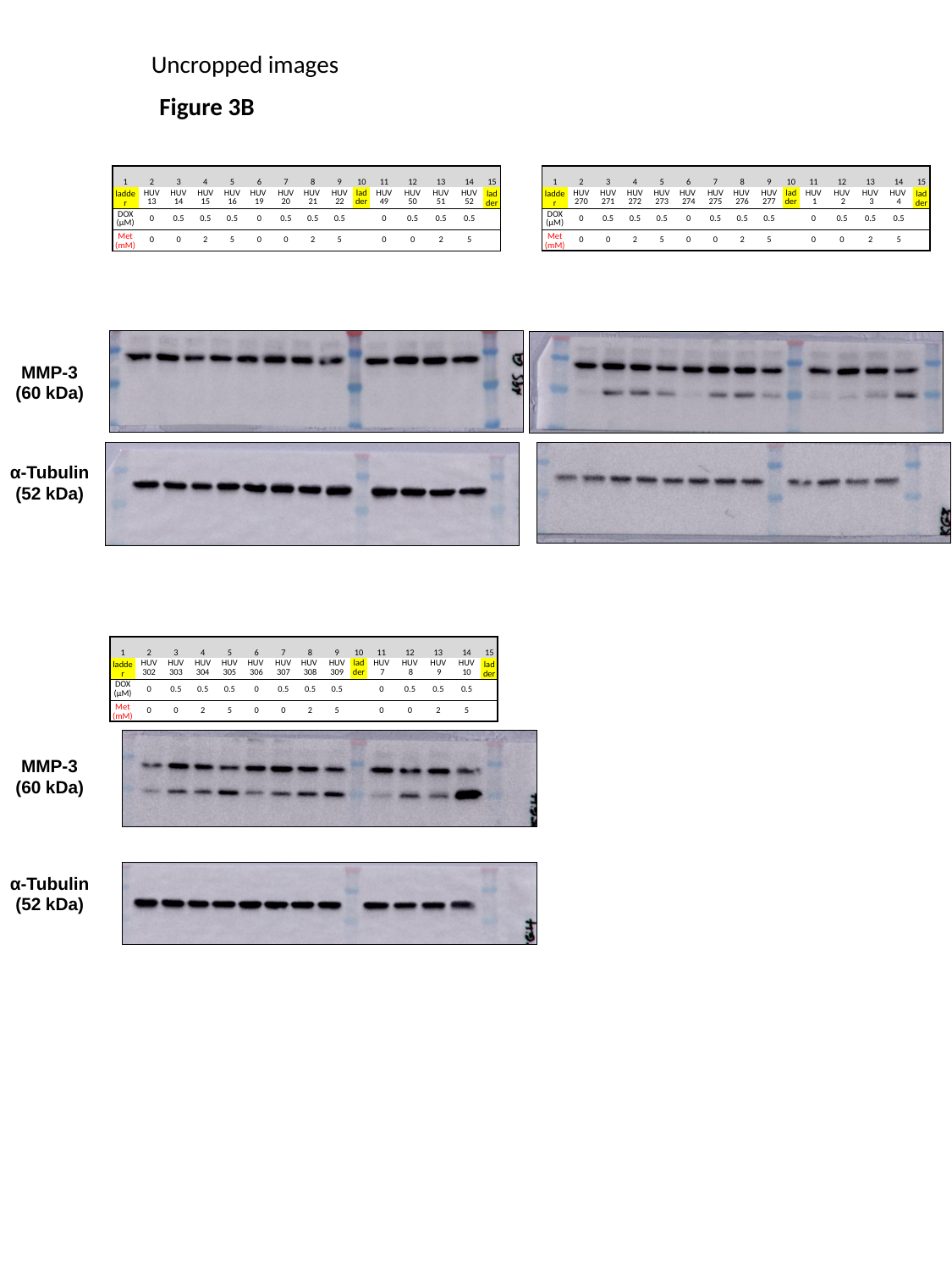

Uncropped images
Figure 3B
| 1 | 2 | 3 | 4 | 5 | 6 | 7 | 8 | 9 | 10 | 11 | 12 | 13 | 14 | 15 |
| --- | --- | --- | --- | --- | --- | --- | --- | --- | --- | --- | --- | --- | --- | --- |
| ladder | HUV 13 | HUV 14 | HUV 15 | HUV 16 | HUV 19 | HUV 20 | HUV 21 | HUV 22 | ladder | HUV 49 | HUV 50 | HUV 51 | HUV 52 | ladder |
| DOX (µM) | 0 | 0.5 | 0.5 | 0.5 | 0 | 0.5 | 0.5 | 0.5 | | 0 | 0.5 | 0.5 | 0.5 | |
| Met (mM) | 0 | 0 | 2 | 5 | 0 | 0 | 2 | 5 | | 0 | 0 | 2 | 5 | |
| 1 | 2 | 3 | 4 | 5 | 6 | 7 | 8 | 9 | 10 | 11 | 12 | 13 | 14 | 15 |
| --- | --- | --- | --- | --- | --- | --- | --- | --- | --- | --- | --- | --- | --- | --- |
| ladder | HUV 270 | HUV 271 | HUV 272 | HUV 273 | HUV 274 | HUV 275 | HUV 276 | HUV 277 | ladder | HUV 1 | HUV 2 | HUV 3 | HUV 4 | ladder |
| DOX (µM) | 0 | 0.5 | 0.5 | 0.5 | 0 | 0.5 | 0.5 | 0.5 | | 0 | 0.5 | 0.5 | 0.5 | |
| Met (mM) | 0 | 0 | 2 | 5 | 0 | 0 | 2 | 5 | | 0 | 0 | 2 | 5 | |
MMP-3
(60 kDa)
α-Tubulin
(52 kDa)
| 1 | 2 | 3 | 4 | 5 | 6 | 7 | 8 | 9 | 10 | 11 | 12 | 13 | 14 | 15 |
| --- | --- | --- | --- | --- | --- | --- | --- | --- | --- | --- | --- | --- | --- | --- |
| ladder | HUV 302 | HUV 303 | HUV 304 | HUV 305 | HUV 306 | HUV 307 | HUV 308 | HUV 309 | ladder | HUV 7 | HUV 8 | HUV 9 | HUV 10 | ladder |
| DOX (µM) | 0 | 0.5 | 0.5 | 0.5 | 0 | 0.5 | 0.5 | 0.5 | | 0 | 0.5 | 0.5 | 0.5 | |
| Met (mM) | 0 | 0 | 2 | 5 | 0 | 0 | 2 | 5 | | 0 | 0 | 2 | 5 | |
MMP-3
(60 kDa)
α-Tubulin
(52 kDa)

## Slide 7
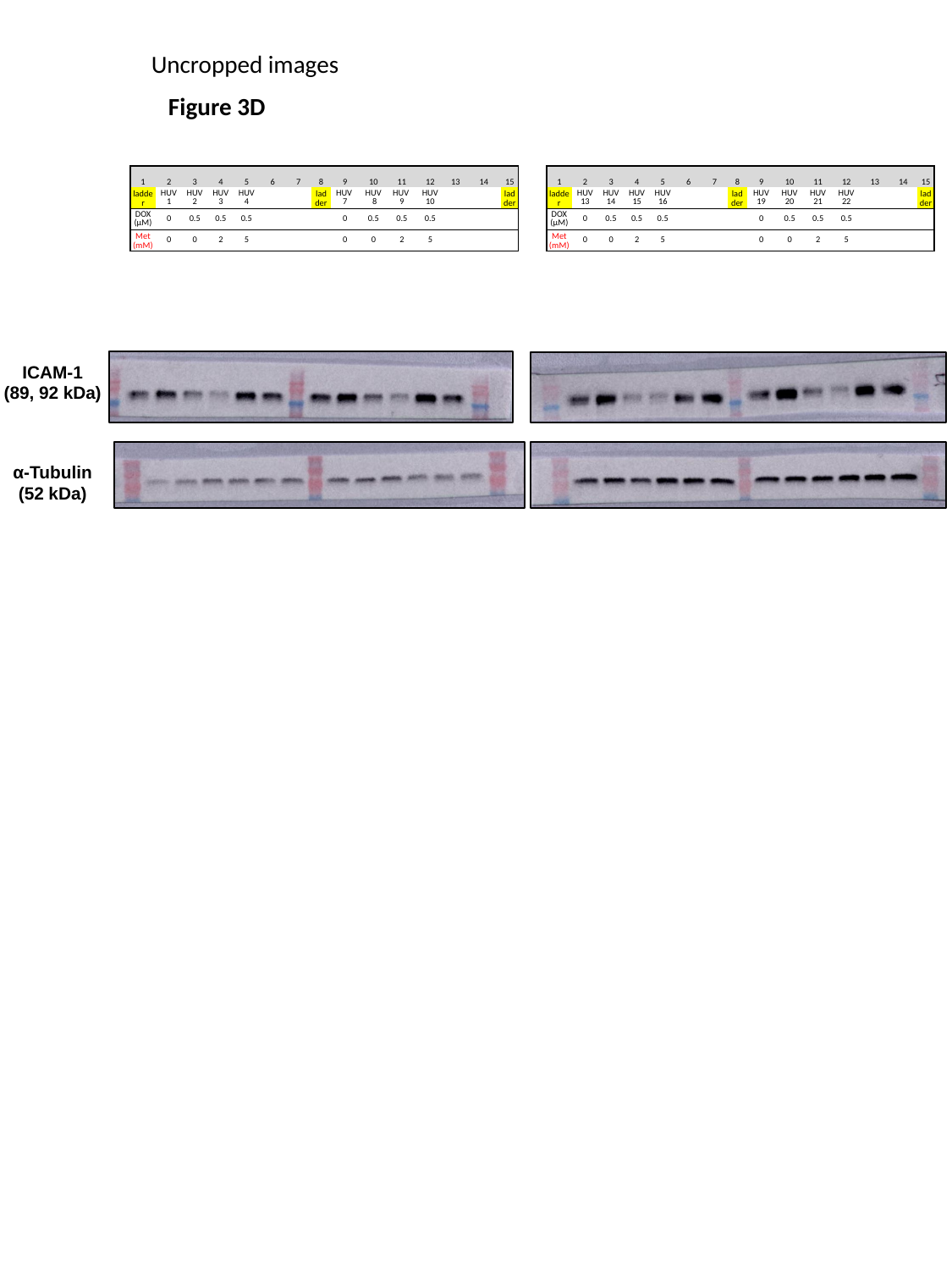

Uncropped images
Figure 3D
| 1 | 2 | 3 | 4 | 5 | 6 | 7 | 8 | 9 | 10 | 11 | 12 | 13 | 14 | 15 |
| --- | --- | --- | --- | --- | --- | --- | --- | --- | --- | --- | --- | --- | --- | --- |
| ladder | HUV 1 | HUV 2 | HUV 3 | HUV 4 | | | ladder | HUV 7 | HUV 8 | HUV 9 | HUV 10 | | | ladder |
| DOX (µM) | 0 | 0.5 | 0.5 | 0.5 | | | | 0 | 0.5 | 0.5 | 0.5 | | | |
| Met (mM) | 0 | 0 | 2 | 5 | | | | 0 | 0 | 2 | 5 | | | |
| 1 | 2 | 3 | 4 | 5 | 6 | 7 | 8 | 9 | 10 | 11 | 12 | 13 | 14 | 15 |
| --- | --- | --- | --- | --- | --- | --- | --- | --- | --- | --- | --- | --- | --- | --- |
| ladder | HUV 13 | HUV 14 | HUV 15 | HUV 16 | | | ladder | HUV 19 | HUV 20 | HUV 21 | HUV 22 | | | ladder |
| DOX (µM) | 0 | 0.5 | 0.5 | 0.5 | | | | 0 | 0.5 | 0.5 | 0.5 | | | |
| Met (mM) | 0 | 0 | 2 | 5 | | | | 0 | 0 | 2 | 5 | | | |
ICAM-1
(89, 92 kDa)
α-Tubulin
(52 kDa)

## Slide 8
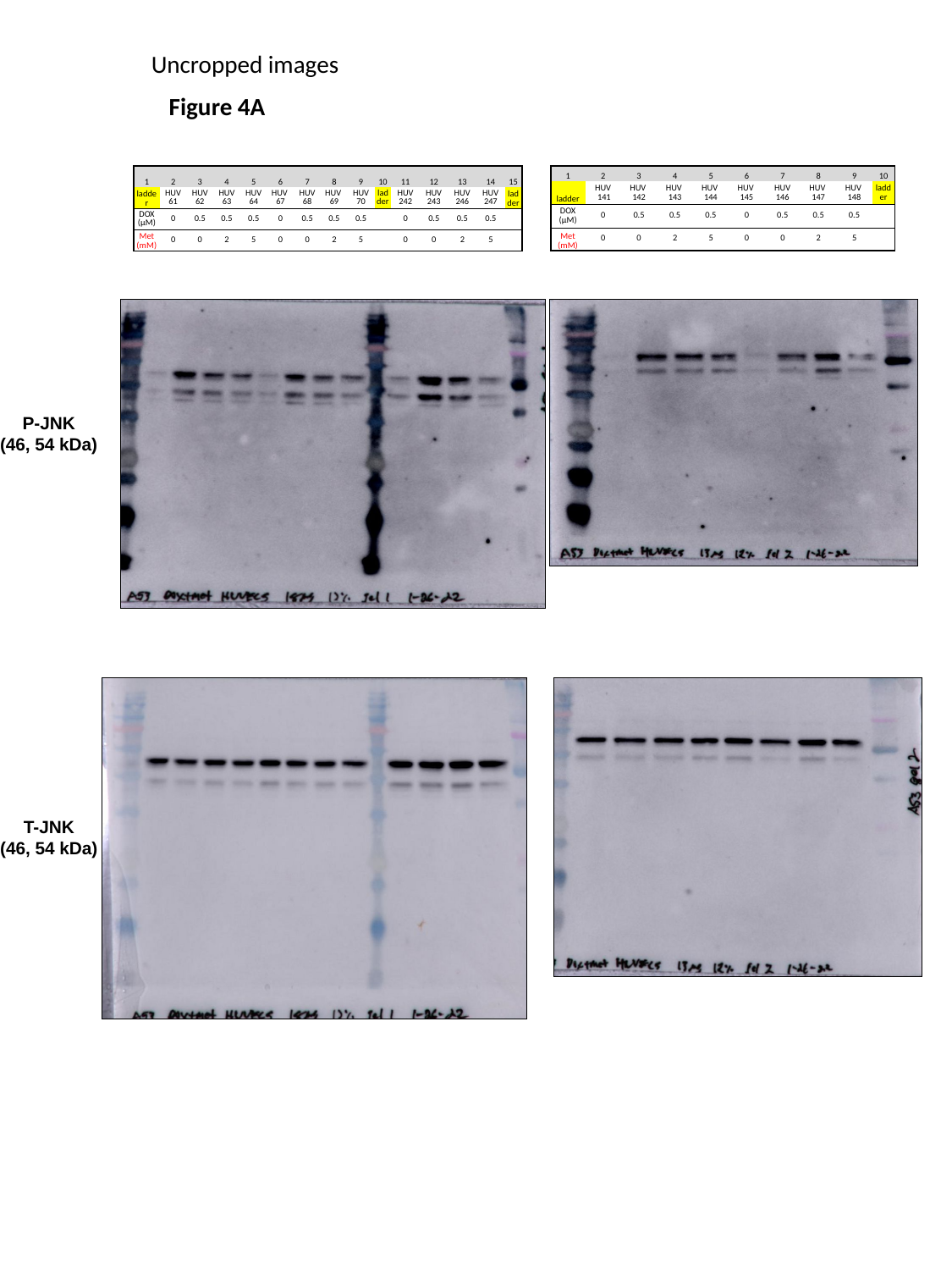

Uncropped images
Figure 4A
| 1 | 2 | 3 | 4 | 5 | 6 | 7 | 8 | 9 | 10 | 11 | 12 | 13 | 14 | 15 |
| --- | --- | --- | --- | --- | --- | --- | --- | --- | --- | --- | --- | --- | --- | --- |
| ladder | HUV 61 | HUV 62 | HUV 63 | HUV 64 | HUV 67 | HUV 68 | HUV 69 | HUV 70 | ladder | HUV 242 | HUV 243 | HUV 246 | HUV 247 | ladder |
| DOX (µM) | 0 | 0.5 | 0.5 | 0.5 | 0 | 0.5 | 0.5 | 0.5 | | 0 | 0.5 | 0.5 | 0.5 | |
| Met (mM) | 0 | 0 | 2 | 5 | 0 | 0 | 2 | 5 | | 0 | 0 | 2 | 5 | |
| 1 | 2 | 3 | 4 | 5 | 6 | 7 | 8 | 9 | 10 |
| --- | --- | --- | --- | --- | --- | --- | --- | --- | --- |
| ladder | HUV 141 | HUV 142 | HUV 143 | HUV 144 | HUV 145 | HUV 146 | HUV 147 | HUV 148 | ladder |
| DOX (µM) | 0 | 0.5 | 0.5 | 0.5 | 0 | 0.5 | 0.5 | 0.5 | |
| Met (mM) | 0 | 0 | 2 | 5 | 0 | 0 | 2 | 5 | |
P-JNK
(46, 54 kDa)
T-JNK
(46, 54 kDa)

## Slide 9
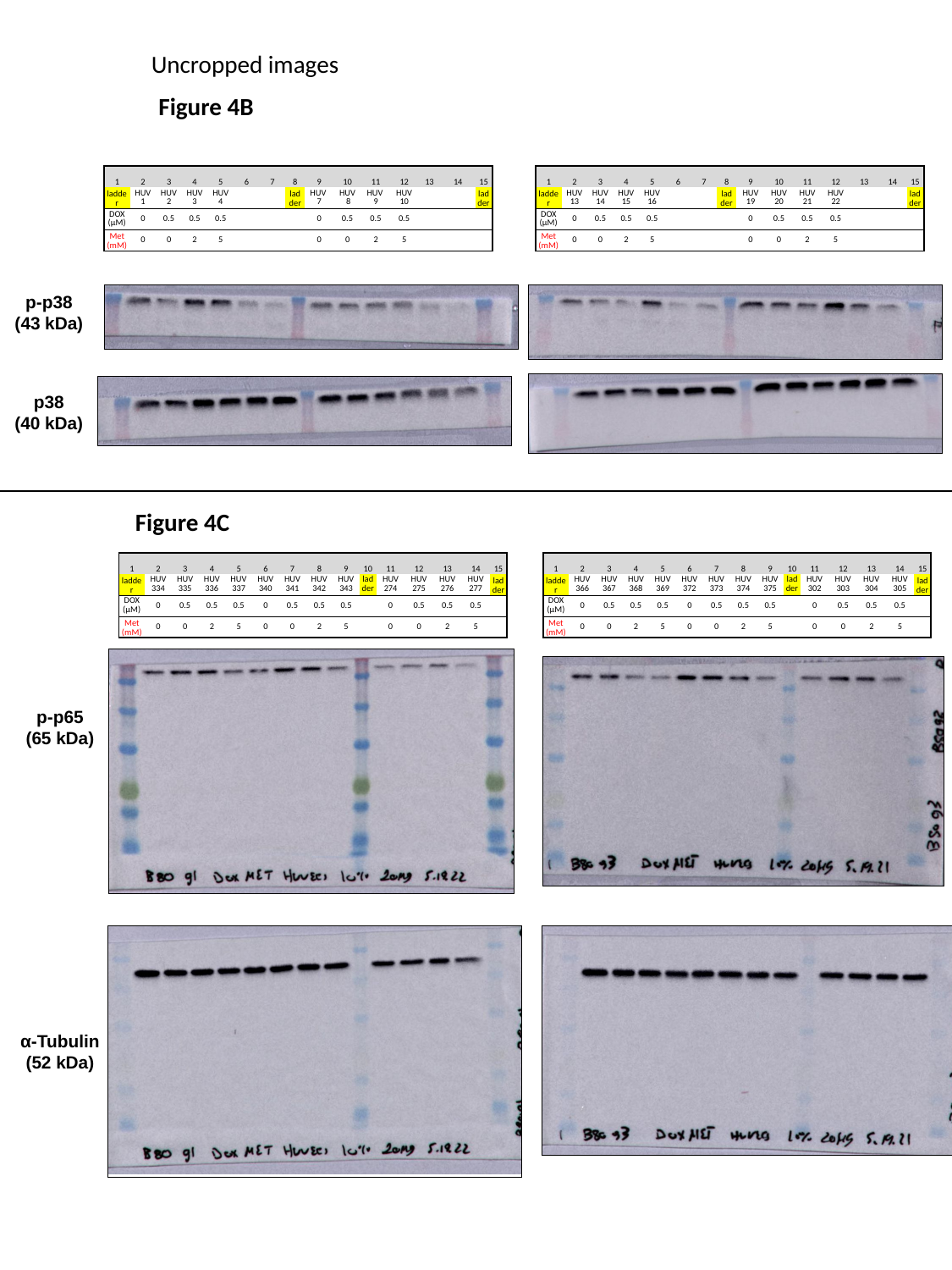

Uncropped images
Figure 4B
| 1 | 2 | 3 | 4 | 5 | 6 | 7 | 8 | 9 | 10 | 11 | 12 | 13 | 14 | 15 |
| --- | --- | --- | --- | --- | --- | --- | --- | --- | --- | --- | --- | --- | --- | --- |
| ladder | HUV 1 | HUV 2 | HUV 3 | HUV 4 | | | ladder | HUV 7 | HUV 8 | HUV 9 | HUV 10 | | | ladder |
| DOX (µM) | 0 | 0.5 | 0.5 | 0.5 | | | | 0 | 0.5 | 0.5 | 0.5 | | | |
| Met (mM) | 0 | 0 | 2 | 5 | | | | 0 | 0 | 2 | 5 | | | |
| 1 | 2 | 3 | 4 | 5 | 6 | 7 | 8 | 9 | 10 | 11 | 12 | 13 | 14 | 15 |
| --- | --- | --- | --- | --- | --- | --- | --- | --- | --- | --- | --- | --- | --- | --- |
| ladder | HUV 13 | HUV 14 | HUV 15 | HUV 16 | | | ladder | HUV 19 | HUV 20 | HUV 21 | HUV 22 | | | ladder |
| DOX (µM) | 0 | 0.5 | 0.5 | 0.5 | | | | 0 | 0.5 | 0.5 | 0.5 | | | |
| Met (mM) | 0 | 0 | 2 | 5 | | | | 0 | 0 | 2 | 5 | | | |
p-p38
(43 kDa)
p38
(40 kDa)
Figure 4C
| 1 | 2 | 3 | 4 | 5 | 6 | 7 | 8 | 9 | 10 | 11 | 12 | 13 | 14 | 15 |
| --- | --- | --- | --- | --- | --- | --- | --- | --- | --- | --- | --- | --- | --- | --- |
| ladder | HUV 334 | HUV 335 | HUV 336 | HUV 337 | HUV 340 | HUV 341 | HUV 342 | HUV 343 | ladder | HUV 274 | HUV 275 | HUV 276 | HUV 277 | ladder |
| DOX (µM) | 0 | 0.5 | 0.5 | 0.5 | 0 | 0.5 | 0.5 | 0.5 | | 0 | 0.5 | 0.5 | 0.5 | |
| Met (mM) | 0 | 0 | 2 | 5 | 0 | 0 | 2 | 5 | | 0 | 0 | 2 | 5 | |
| 1 | 2 | 3 | 4 | 5 | 6 | 7 | 8 | 9 | 10 | 11 | 12 | 13 | 14 | 15 |
| --- | --- | --- | --- | --- | --- | --- | --- | --- | --- | --- | --- | --- | --- | --- |
| ladder | HUV 366 | HUV 367 | HUV 368 | HUV 369 | HUV 372 | HUV 373 | HUV 374 | HUV 375 | ladder | HUV 302 | HUV 303 | HUV 304 | HUV 305 | ladder |
| DOX (µM) | 0 | 0.5 | 0.5 | 0.5 | 0 | 0.5 | 0.5 | 0.5 | | 0 | 0.5 | 0.5 | 0.5 | |
| Met (mM) | 0 | 0 | 2 | 5 | 0 | 0 | 2 | 5 | | 0 | 0 | 2 | 5 | |
p-p65
(65 kDa)
α-Tubulin
(52 kDa)

## Slide 10
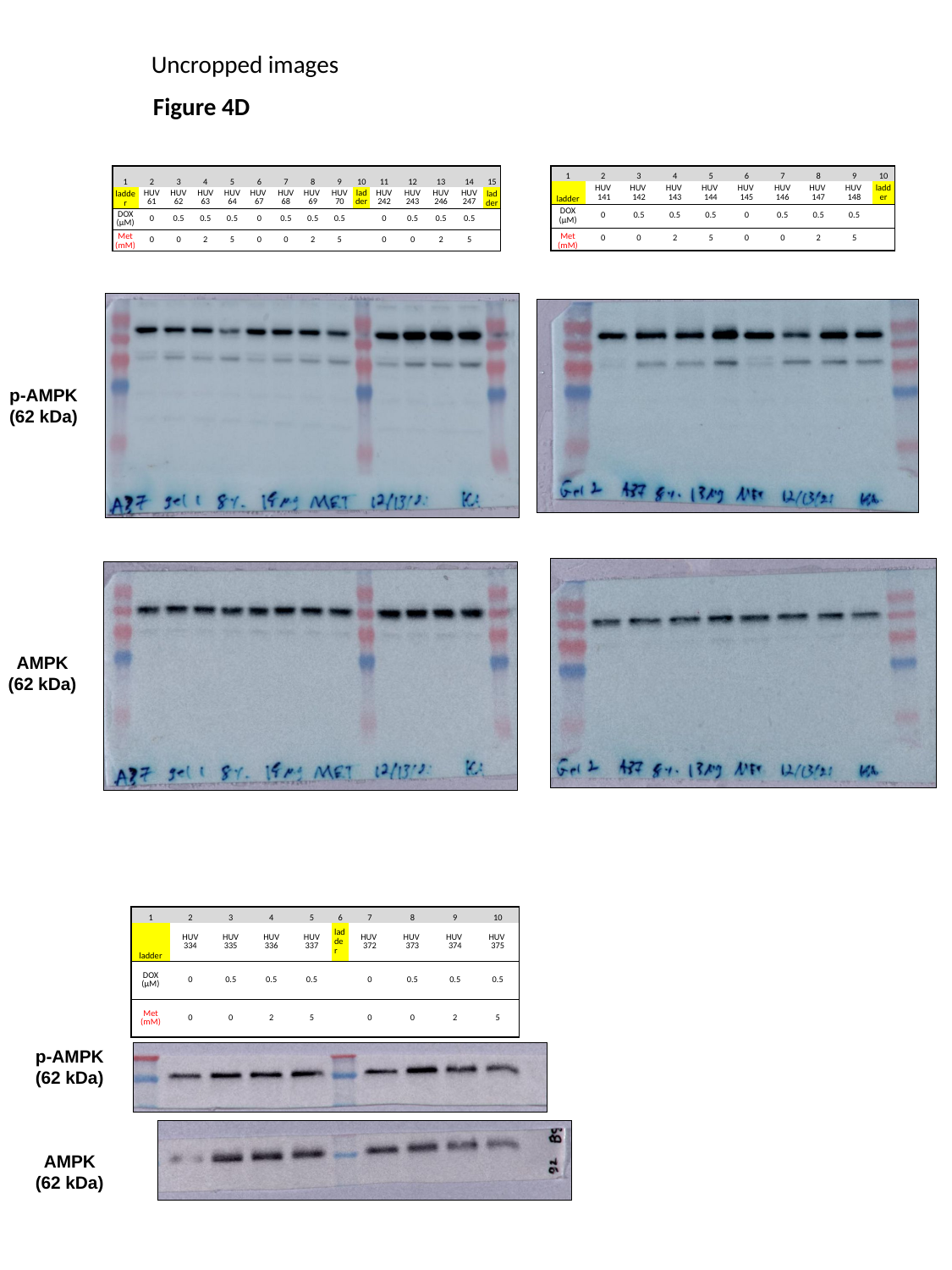

Uncropped images
Figure 4D
| 1 | 2 | 3 | 4 | 5 | 6 | 7 | 8 | 9 | 10 | 11 | 12 | 13 | 14 | 15 |
| --- | --- | --- | --- | --- | --- | --- | --- | --- | --- | --- | --- | --- | --- | --- |
| ladder | HUV 61 | HUV 62 | HUV 63 | HUV 64 | HUV 67 | HUV 68 | HUV 69 | HUV 70 | ladder | HUV 242 | HUV 243 | HUV 246 | HUV 247 | ladder |
| DOX (µM) | 0 | 0.5 | 0.5 | 0.5 | 0 | 0.5 | 0.5 | 0.5 | | 0 | 0.5 | 0.5 | 0.5 | |
| Met (mM) | 0 | 0 | 2 | 5 | 0 | 0 | 2 | 5 | | 0 | 0 | 2 | 5 | |
| 1 | 2 | 3 | 4 | 5 | 6 | 7 | 8 | 9 | 10 |
| --- | --- | --- | --- | --- | --- | --- | --- | --- | --- |
| ladder | HUV 141 | HUV 142 | HUV 143 | HUV 144 | HUV 145 | HUV 146 | HUV 147 | HUV 148 | ladder |
| DOX (µM) | 0 | 0.5 | 0.5 | 0.5 | 0 | 0.5 | 0.5 | 0.5 | |
| Met (mM) | 0 | 0 | 2 | 5 | 0 | 0 | 2 | 5 | |
p-AMPK
(62 kDa)
AMPK
(62 kDa)
| 1 | 2 | 3 | 4 | 5 | 6 | 7 | 8 | 9 | 10 |
| --- | --- | --- | --- | --- | --- | --- | --- | --- | --- |
| ladder | HUV 334 | HUV 335 | HUV 336 | HUV 337 | ladder | HUV 372 | HUV 373 | HUV 374 | HUV 375 |
| DOX (µM) | 0 | 0.5 | 0.5 | 0.5 | | 0 | 0.5 | 0.5 | 0.5 |
| Met (mM) | 0 | 0 | 2 | 5 | | 0 | 0 | 2 | 5 |
p-AMPK
(62 kDa)
AMPK
(62 kDa)

## Slide 11
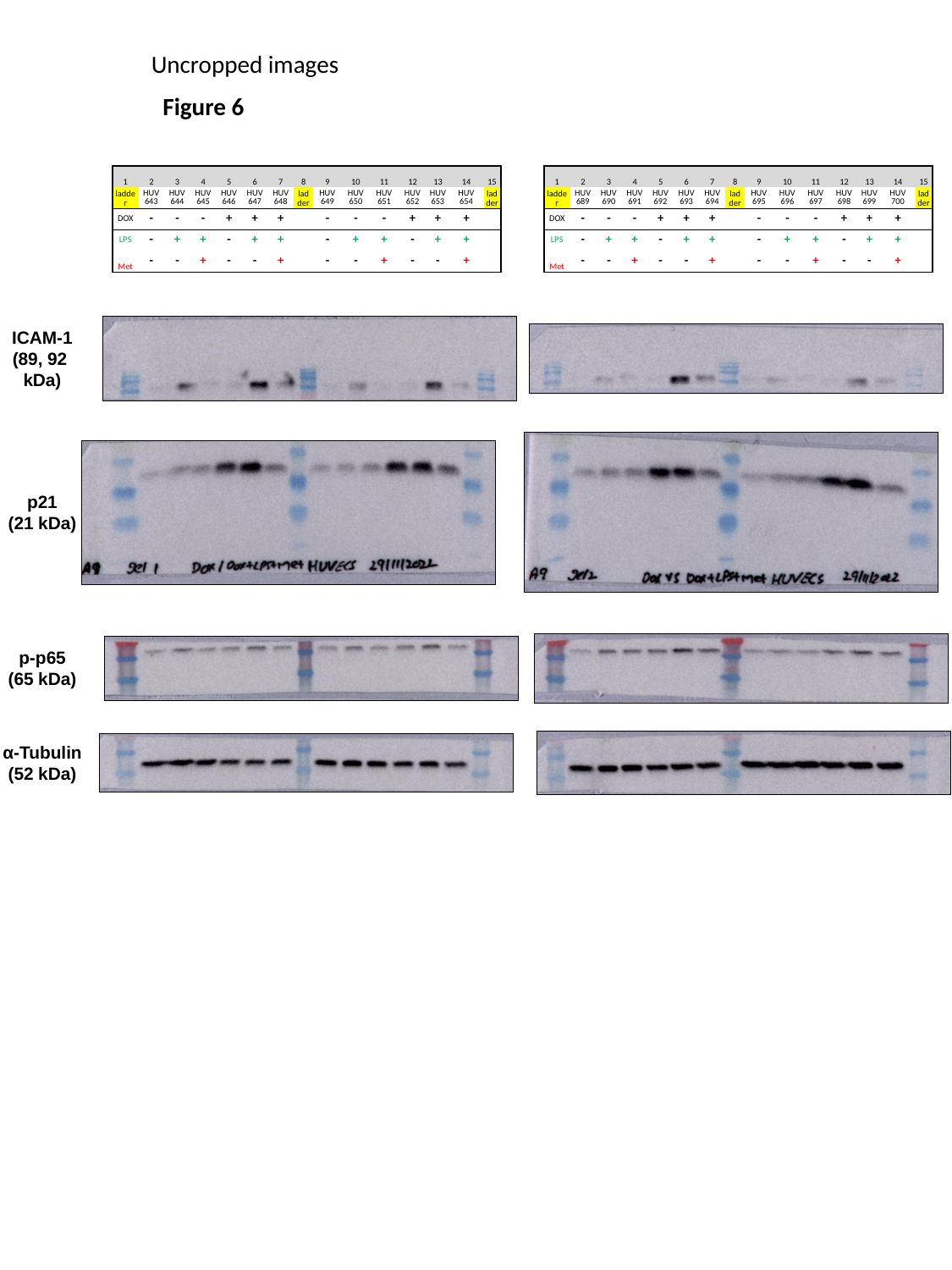

Uncropped images
Figure 6
| 1 | 2 | 3 | 4 | 5 | 6 | 7 | 8 | 9 | 10 | 11 | 12 | 13 | 14 | 15 |
| --- | --- | --- | --- | --- | --- | --- | --- | --- | --- | --- | --- | --- | --- | --- |
| ladder | HUV 689 | HUV 690 | HUV 691 | HUV 692 | HUV 693 | HUV 694 | ladder | HUV 695 | HUV 696 | HUV 697 | HUV 698 | HUV 699 | HUV 700 | ladder |
| DOX | - | - | - | + | + | + | | - | - | - | + | + | + | |
| LPS | - | + | + | - | + | + | | - | + | + | - | + | + | |
| Met | - | - | + | - | - | + | | - | - | + | - | - | + | |
| 1 | 2 | 3 | 4 | 5 | 6 | 7 | 8 | 9 | 10 | 11 | 12 | 13 | 14 | 15 |
| --- | --- | --- | --- | --- | --- | --- | --- | --- | --- | --- | --- | --- | --- | --- |
| ladder | HUV 643 | HUV 644 | HUV 645 | HUV 646 | HUV 647 | HUV 648 | ladder | HUV 649 | HUV 650 | HUV 651 | HUV 652 | HUV 653 | HUV 654 | ladder |
| DOX | - | - | - | + | + | + | | - | - | - | + | + | + | |
| LPS | - | + | + | - | + | + | | - | + | + | - | + | + | |
| Met | - | - | + | - | - | + | | - | - | + | - | - | + | |
ICAM-1
(89, 92
kDa)
p21
(21 kDa)
p-p65
(65 kDa)
α-Tubulin
(52 kDa)
